# ALL-IN meta-analysis: breathing life into living systematic reviews

**DOI:** 10.12688/f1000research.74223.1

**Published:** 2022-05-19

**Authors:** Judith ter Schure, Peter Grünwald

**Affiliations:** 1 Machine Learning, CWI, Amsterdam, The Netherlands; 2 Mathematics, Leiden University, Leiden, The Netherlands

**Keywords:** Anytime, Live, Leading, Interim, Meta-analysis, Efficiency, Collaboration, Communication, Research Waste

## Abstract

Science is justly admired as a cumulative process (“standing on the shoulders of giants”), yet scientific knowledge is typically built on a patchwork of research contributions without much coordination. This lack of efficiency has specifically been addressed in clinical research by recommendations for living systematic reviews and against research waste. We propose to further those recommendations with ALL-IN meta-analysis: Anytime Live and Leading INterim meta-analysis. ALL-IN provides statistical methodology for a meta-analysis that can be updated at any time—reanalyzing after each new observation while retaining type-I error guarantees, live—no need to prespecify the looks, and leading—in the decisions on whether individual studies should be initiated, stopped or expanded, the meta-analysis can be the leading source of information. We illustrate the method for time-to-event data, showing how synthesizing data at interim stages of studies can increase efficiency when studies are slow in themselves to provide the necessary number of events for completion. The meta-analysis can be performed on interim data, but does not have to. The analysis design requires no information about the number of patients in trials or the number of trials eventually included. So it can breathe life into living systematic reviews, through better and simpler statistics, efficiency, collaboration and communication

The scientific response to the coronavirus disease 2019 (Covid-19) pandemic constitutes a major gamble. In the United States, for example, the funding program for vaccine development did not put money on a single vaccine, but on six different ones. They purposely took “multiple shots on goal” according to Larry Corey of the National Institutes of Health (NIH) Covid-19 Prevention Network in an interview with STAT ( Branswell, 2021). Vaccine development is not a sure thing, and so their strategy needed to be robust enough to just “let the chips fall”. Also in the search for treatments, the scientific community had to hedge its bets. Clinical trials competed for resources and patients, and had to continuously change course when new information arrived. In contrast to vaccines, however, in most countries a strategy to find treatments was lacking. Many clinical trials suffered from “poor questions, poor study design, inefficiency of regulation and conduct, and non or poor reporting of results”: research waste ( Glasziou *et al*., 2020). We believe that more strategic thinking can benefit a future pandemic response as well as non-pandemic evidence-based medicine, as uncertainty is often a given. Honest scientific bets can breathe life into the approach called *living systematic reviews* that aims to keep the evidence record up-to-date ( Elliott *et al*., 2017) and the medical guidelines current ( Akl *et al*., 2017). We propose to make those bets by using *ALL-IN meta-analysis* in clinical trial design, monitoring and reporting.

ALL-IN meta-analysis stands for *Anytime Live* and *Leading INterim* meta-analysis. The *Anytime* aspect provides analysis that controls type-I error in testing and coverage in interval estimation regardless of the decision making along the way, and so regardless of any stopping rules or accumulation bias processes ( Ter Schure & Grünwald, 2019). The *Live* aspect prevents research waste caused by meta-analyses that are out-of-date, which is often the case in retrospective meta-analysis. The synthesis can be a bottom-up collaboration of trials, as well as a prospective top-down statistical analysis for decision making. The *Leading* aspect allows the systematically collected evidence included so far to drive the necessity and design of new trials. Finally, the *INterim* aspect is new in meta-analysis and makes for effortless combination of trials while they are still ongoing. What is more, ALL-IN meta-analysis is also literally *ALL-IN* since any number of new studies can be included; it has an unlimited horizon. Like the use of the law of iterated logarithm in meta-analysis, proposed by Lan *et al*., 2003, Hu *et al*., 2007, this property goes back to early work by Robbins and colleagues ( Robbins, 1970). We illustrate this in the setting of time-to-event data, where waiting for new events is an inherent challenge of clinical trials. Combining trials early can prevent delays if studies are slow in themselves to complete the necessary number of events. ALL-IN has advantages in four categories: statistics, efficiency, collaboration and communication. We introduce all four briefly (page 4–5) before we go into more detail, but first illustrate the language of betting for single trials studying a Covid-19 vaccine.

## A single trial: the FDA game

On June 30th, 2020, the US Food and Drug Administration (FDA) published its guidance document on “Development and Licensure of Vaccines to Prevent Covid-19” ( FDA, 2020). This set the goals for any Phase III clinical trial betting on a protective effect of a vaccine against Covid-19. The guidance document advised on the definition of events of confirmed (symptomatic) SARS-CoV-2 infection for the trials to be counting. And in counting those, the document prescribed the two things to achieve: (1) at least a vaccine efficacy (VE) of 50% and (2) evidence against a null hypothesis of ≤ 30% VE ( FDA, 2020, p. 14). Most Covid-19 vaccine trials randomized large numbers of participants 50:50 vaccine:placebo such that we can assume that also throughout the trial the participants at risk stayed approximately balanced. According to the definition of SARS-Cov-2 infections, we start counting once a participant has a confirmed infection after being fully vaccinated for at at least a number of days, e.g. 7 days in the Pfizer-BioNTech trial ( Polack *et al*., 2020). This is also when a (virtual) bet could start. In the following we reinterpret the design for the Covid-19 vaccine trials in the language of betting.

Each new event carries evidence that we express by a betting score. We make a (virtual) investment on one of the two outcomes: either the next event occurs in the vaccine group or it occurs in the placebo group. If there is no effect of the vaccine whatsoever, the 50:50 risk set ensures that the infected participant has 0.5 a chance to be vaccinated and 0.5 a chance to be a placebo. Yet, following the FDA, we do not only want to rule out an ineffective vaccine, but also reject the hypothesis that the vaccine has an effect that is too small—set as the null hypothesis of (at most) 30% VE. In that case each newly observed infection has slightly smaller chance to be a vaccinated participant. That probability to be in the vaccine group is 0.41, since each placebo group member has a 100% risk of Covid-19 and a vaccine group member has 100−30 = 70% of the risk, which is a fraction 0.41 of the total risk (70/(100 + 70)). So if the VE is too small to be of interest we expect (at least) a fraction 0.41 of Covid-19 events to occur in the vaccine group and (at most) 0.59 in placebo.

How do we bet against that and win if the vaccine has a much larger protective effect? We are betting *against* the probability 0.41 of the next Covid-19 event to occur in the vaccine group. If this probability actually is that large (the vaccine is not very protective; the null hypothesis) we do not want the game to be favorable under any strategy, just like the casino does not want any gambler to earn a salary playing the roulette wheel. On the other hand, we are betting *in favor* of a much smaller probability for the vaccine group. If this probability is smaller (the vaccine is protective; the alternative hypothesis) we do want to win money, just like a professional poker player who makes a salary out of gambling well. We use the betting scores to decide whether the vaccine is a real deal-breaker (the scores behave like the salary of a professional poker player) or whether it is not effective enough (the scores behave like anyone playing the roulette wheel). To ensure that our betting scores can show either case, we first *design* the game such that it is fair—under the null hypothesis—and then *optimize playing* the game with a strategy that is profitable —under the alternative.

**Designing a fair game under the null hypothesis** Consider gambling at the roulette table where the vaccine trial analogy is like betting on red (vaccine) or black (placebo). Betting correctly doubles your investment, betting incorrectly loses everything you risked. Assuming no house edge (no 0 or 00 on the roulette wheel) and an initial €100 you do not expect to increase your investment, since you have 0.5 a chance of doubling (2 · €100) and 0.5 a chance of losing all (0 · €100). Whether you bet everything on black or red, in expectation the betting score after one round is (0.5·2+0.5·0)·€100, which is the initial investment €100. To achieve the same thing betting against the 0.41:0.59 probabilities instead of 0.5:0.5, your investment needs to multiply by 2.4 (1/0.41) for vaccine and 1.7 (1/0.59) for placebo. If you bet everything on vaccine you have 0.41 chance of multiplying by 2.4 (2.4 · €100) and 0.59 chance of losing all (0 · €100) and if you bet everything on placebo you have 0.59 chance of multiplying by 1.7 (1.7·€100) and 0.41 chance of losing all (0·€100). The expected betting score after one round is again the initial investment for both: (0.41·1/0.41+0.59·0)·€100 and (0.59 · 1/0.59 +0.41 · 0) · €100. Hence, at either the roulette table or in this FDA game, by design the game is fair and does not favor us. After all, if our observed infections land on the vaccine and control group with the probabilities 0.41:0.59, like a spin of the roulette wheel on black and red with 0.5:0.5, we do not expect to claim an effective vaccine.

**Optimize playing the game under the alternative hypothesis** How do we win as fast and as much as possible if our observed infections do not behave like a roulette wheel? It has been known since the work of Kelly (1956) and Breiman (1961) that the best way to increase your capital in the long run is to not bet all your (virtual) investment €100 on one of the two possible outcomes (red/vaccine or black/placebo) but to divide it based on the odds that make the game favorable to you. So our focus needs to be on the minimal VE of 50% from the FDA guidance. In the scenario of 50% VE, the probability that the next Covid-19 case is in the vaccine group is 1/3: if we set the risk of Covid-19 for a placebo group member to 100%, a vaccine group member has 100−50 = 50% of that risk, which is 1/3 of the total risk (50/(100 + 50)). Kelly (1956) and Breiman (1961) urge us to invest one-third (1/3 · €100) on observing the next infection in the vaccine group and two-thirds (2/3 · €100) on placebo.

**Likelihood ratios** If we bet this way we can rewrite our betting scores in terms of a *likelihood ratio*. We first show this for the red-black roulette game where we double what we had put at risk on either black or red if the spin of the roulette wheel outputs the color we bet on. Just like in our strategy in the FDA game, we put 1/3 · €100 on red and 2/3 · €100 on black, so we win the following if the ball *X* lands on either **red** or **black**:


$$\begin{array}{l} X=red\;2\text{⋅}\frac{1}{3}\text{⋅}€100=\frac{L(1/\text{3}|X)}{L(1/\text{2}|X)}\text{⋅}€100\\ X=black\;2\text{⋅}\frac{2}{3}\text{⋅}€100=\frac{L(1/\text{3}|X)}{L(1/\text{2}|X)}\text{⋅}€100 \end{array}$$

The Bernoulli 1/3-likelihood (1/3 | *X*) assigns likelihood 1/3 when is *X* = **red** and 2/3 when is *X* = **black**. So if our strategy is to invest 1/3-2/3 in roulette, our payout is our initial investment €100 multiplied by the likelihood ratio, whether *X* is **red** or **black**.


$$\begin{array}{l} X=vaccinated\;2.4\text{⋅}\frac{1}{3}\text{⋅}€100=\frac{L(50\%\;\text{VE}|X)}{L(30\%\;\text{VE}|X)}\text{⋅}€100\\ X=placebo\;1.7\text{⋅}\frac{2}{3}\text{⋅}€100=\frac{L(50\%\;\text{VE}|X)}{L(30\%\;\text{VE}|X)}\text{⋅}€100 \end{array}$$

The likelihood for 50% VE ((50% VE | *X*)) assigns likelihood 1/3 when is *X* = **vaccine** and 2/3 when is *X* = **placebo**. Similarly, the likelihood for 30% VE ((30% VE | *X*)) assigns likelihood 0.41 when is *X* = **vaccine** and 0.59 when is *X* = **placebo**. Hence if our strategy is to invest 1/3-2/3 in the FDA game, our payout is also our initial investment €100 multiplied by the likelihood ratio, whether *X* is **vaccine** or **placebo**.

**Reinvesting** We assume now that we start with an initial (virtual) investment of €1 instead of €100, to easily assess our winnings by the factor with which we multiply our initial investment. At the first observation we bet €0.33 on vaccine and €0.66 on placebo. After we observe the event in the placebo group we lose our €0.33 bet on vaccine and multiply our €0.66 on placebo by 1.7 to €1.13. The likelihood ratio between our 30% VE alternative hypothesis and our 50% VE null hypothesis— so (50% VE | *X* )/(30% VE | *X* )—is also about 1.13, so multiplying our initial investment of €1 into €1.13. On the other hand, if we observe the event in the vaccine group we lose our €0.66 bet on a placebo event and multiply our €0.33 on vaccine by 2.4 to €0.81. The likelihood ratio of a vaccine event multiplies our investment by 0.81. After each observed event we reinvest what we have left in the new bet, so multiply that with the next likelihood ratio.

**A winner** The Pfizer/BioNTech trial observed 8 cases of Covid-19 among participants assigned to receive the vaccine and 162 cases among those assigned to placebo ( Polack *et al*., 2020). This totals a betting score of 0.81 ^8^ · 1.13 ^162^· €1, which is about €118 million (note that 1.13 is really 1.13333 . . .). If someone wins that at the poker table, we have good reason to consider her a professional poker player with a favorable strategy, rather than a lucky beginner ( Konnikova, 2020).

## Meta-analysis: bottom-up collaboration

The Pfizer/BioNTech trial included more than 43 thousand participants ( Polack *et al*., 2020), which is quite unique for a clinical trial. Usually trials are much smaller, and scientific consensus is built through systematic reviews and retrospectively combining trials in a meta-analysis. ALL-IN is a way to do so by collaborating bottom-up in a strategic way that can be live instead of retrospective. It has advantages in four categories that we will first briefly introduce and then further elaborate on in this paper: statistics, efficiency, collaboration and communication.

### Statistics

Not all mRNA vaccines showed such favourable results as the Pfizer/BioNTech vaccine. In a press release CureVac AG (2021) announced that the final analysis of their clinical trial observed 83 events in the vaccinated group and 145 in placebo, so only a 43% VE (our calculations assuming a 50:50 balanced risk set ( *r* = 1 in CureVac AG (2020, p. 124)) ^1^). Their protocol states the FDA goal in terms of a confidence interval that excludes a VE of 30%, adjusted for two interim analyses. That adjusted confidence interval at the final analysis is based on *Z*_*α*/2_-statistic for the nominal level *α*/2 = 0.02281 ( CureVac AG, 2020, Table 8). That interval is [25.3%, 57.1% VE] (our calculations; normal approximation interval) and, regrettably, does not exclude 30%. When the chips fell, this trial lost.

Statistical analyses like these are essentially *all-or-nothing*, just as any other *p* < *α* analysis. As soon as all the *α* is spent—either on a few interims and a final analysis or just on one fixed sample size—we cannot continue the trial and perform subsequent analyses without violating the type-I error rate. This might be a reasonable price to pay in the urgency of a pandemic when multiple vaccines are competing, but it is a very inconvenient property for clinical trials in general. Usually, we do want to reanalyze a clinical trial in combination with other similar trials in a meta-analysis. Yet any *p* < *α* procedure is equivalent to setting a rejection region for the test statistic and checking whether the value for the statistic falls within that region. This rejection region is based on a sampling distribution that assumes the number of studies in the meta-analysis, and the number of patients within each study to be fixed in advance. Given such a fixed sample size (but also for any sequential stopping rule that sets a maximum sample size in advance, such as *α*-spending), there is only one region, and your test statistic is either in it or not. If it is not, you are not allowed to redo the analyses with an increased sample size. This problem is recognized in approaches to control type-I error for living systematic reviews ( Simmonds *et al*., 2017). But also if the meta-analysis is not updated, the *α* is essentially already spent on the individual trial analyses, since the meta-analysis is an update of the trial analysis that is unscheduled and lacks type-I error control at the same level *α*. If the individual study analysis would have been conclusive, the meta-analysis might never be performed, and we can recognise that we are dealing with a situation of “meta-optional-stopping”. A different way to see this is by the actual sampling distribution of trials in a meta-analysis: any data-driven decision within the series—whether to accumulate more studies and when to perform the meta-analysis—changes the sampling distribution and invalidates the fixed-sample-size distribution assumed for *p* < *α*. Hence hardly any meta-analysis has valid type-I error control, when the accumulation of trials is based on strategic decisions that introduce accumulation bias ( Ter Schure & Grünwald, 2019).

ALL-IN meta-analysis is not *all-or-nothing* but can still combine all available studies. In fact, it allows any number of new studies or patients to be included without ever spending all *α*. In terms of gambling, we can keep betting our virtual investment because we never lose everything. The CureVac AG (2021) results, for example, would have accumulated a betting score of 0.81 ^83^ · 1.13 ^145^· €1 = €1.84. This single trial is not very profitable, but at least it still preserves some evidence to reinvest in the next trial, such that we can continue to observe evidence and express it by betting on additional observations in a new trial. An ALL-IN meta-analysis can always continue testing the null hypothesis—with type-I error control—and estimating the confidence interval—with coverage guarantees. Importantly, for these tests and intervals the procedures are exactly the same no matter what decisions—so-called stopping rules, or accumulation bias processes ( Ter Schure & Grünwald, 2019)—are at play.

### Efficiency

Lack of efficiency has been addressed in clinical research in many ways. Not only in the proposal of living systematic reviews ( Elliott *et al*., 2017), but also in encouragements to present new studies in the context of existing evidence ( Young & Horton, 2005), in advice to design new trials based on systematic reviews and meta-analysis ( Chalmers & Lau, 1993; Goudie *et al*., 2010; Lau *et al*., 1995; Lund *et al*., 2016; Sutton *et al*., 2007) and in pleading to prevent the “scandal” of wasteful research into clinical questions that are already answered or not of primary importance ( Altman, 1994; Chalmers & Glasziou, 2009; Ioannidis *et al*., 2014; Glasziou & Chalmers, 2018; Glasziou *et al*., 2020, “research waste”). These calls have not been completely ignored, since clinical research has seen an increase in efficiency—e.g. in platform trials or adaptive meta-analysis ( Tierney *et al*., 2021)—whenever collaboration is deemed possible prospectively. Nevertheless, most clinical trial data is synthesized retrospectively, and still deserves all of the above recommendations. ALL-IN meta-analysis enables these data-driven decisions that can make science more efficient. New studies can be easily informed by the synthesis of all data so far such that exactly the right number of patients are randomized to answer a research question, no more and no less. Moreover, an ALL-IN meta-analysis can give an account of the evidence at anytime and therefore facilitate prioritizing new studies, if more than one line of research needs additional data, but not all can be funded.

### Collaboration

ALL-IN meta-analysis can be a *live* meta-analysis, since it does not matter how many studies will eventually be combined or which study will contribute most data. Whether it is based on summary statistics ( Tierney *et al*., 2021) or on individual patient data (IPD) ( Polanin & Williams, 2016), involvement in the same meta-analysis facilitates discussion between those running trials in the same line of research; especially if the line of research can be concluded early. Trial protocols and statistical analysis plans can be exchanged and scrutinized, to identify discrepancies between the design and the conduct of trials. In an ongoing meta-analysis, trials can be selected for inclusion before investigators are unblinded to the results, which helps to mitigate the problems of publication bias and *p*-hacking. If IPD analysis is possible, intense collaboration might also prevent mistakes and fraudulent data that would otherwise depreciate the meta-analysis.

A meta-analysis benefits from homogeneity. With too much heterogeneity, it can be very disheartening to update a random-effects meta-analysis, since many trials are needed to precisely estimate the between trial variation and overcome it ( Jackson & Turner, 2017; Kulinskaya & Wood, 2014; Sutton *et al*., 2007). Close collaboration might prevent unnecessary heterogeneity, if trial investigators are involved in the selection of trials in the meta-analysis; especially if they can advise on the design and conduct of new trials and align inclusion criteria and endpoint definitions. A fixed-effects meta-analysis can conclude the research effort early. Sufficient homogeneity may be possible in close collaboration.

### Communication

**The language of betting** The interpretation of evidence in terms of a betting score might help to communicate the uncertainty in statistical results. As Shafer (2021) puts it: “When statistical tests and conclusions are framed as bets, everyone understands their limitations. Great success in betting against probabilities may be the best evidence we can have that the probabilities are wrong, but everyone understands that such success may be mere luck.” Thinking in terms of bets also helps to understand when statistical analyses can be *anytime-valid*. If they are of the *all-or-nothing* kind, but reanalyzed in a meta-analysis, they are gambling while broke. (This intuition can be made mathematically precise; see the description of Neyman-Pearson testing in terms of betting Shafer (2021) and Grünwald *et al*. (2019).) Yet if we add new studies to an ALL-IN meta-analysis, we are reinvesting the betting score that we saved from earlier studies, to evaluate whether the strategy in those earlier studies continues to succeed. Just like when reinvesting your profits in a casino from one slot machine into another, the notion of winning stays the same. Our evidence against the hypothesis of a *fair* casino does not change when we alternate slot machines. It does not change if we use the score so far to decide on alternating them or to decide when to cash out. If the slot machines are fair, any strategy of playing them is not expected to make money, and our notion of type-I error control holds under any dependency on past results (stopping rules or accumulation bias processes).

**Other communication** Those uncomfortable with the language of betting can also easily resort to any of three more familiar notions of statistical communication. Firstly, the likelihood ratios/betting scores and their generalizations, so-called *e-values* ( Grünwald *et al*., 2019; Vovk & Wang, 2021), can be interpreted as conservative *p*-values by taking their inverse. If we denote any betting score or *e*-value by €(e.g. € = €1.84 for the CureVac trial data), then *p* < 1/€ is a conservative *p*-value (e.g. p = 1/1.84 = 0.54 for the CureVac trial data). If we communicate the *p*-value *p* = 1/€ anyone can test by comparing *p* < *α* but with the addition that this conservative *p*-value is anytime valid ^2^ and so *p* < *α* can never spend all *α* (it is never an *all-or-nothing* test). Secondly, the likelihood ratios have their own notion of evidence in the likelihood paradigm ( Royall, 1997). Just as well as stating that the Pfizer/BioNTech trial ( Polack *et al*., 2020) multiplied €1 to almost €118 million and the CureVac AG (2021) trial multiplied €1 to €1.84, we can state that their data was almost 118 million times and 1.84 times more likely if we assume the FDA’s goal of 50% VE in comparison to assuming only 30% VE. For Pfizer, that sounds very good, for CureVac, not so much, and so these numbers have an interpretation of their own without imposing any *α* level. Thirdly, likelihood ratios can be accepted by the Bayesian paradigm, as Bayes factors, and possibly combined with prior odds. Grünwald *et al*. (2019) and Grünwald (2021) show that betting scores/ *e*-values and Bayes factors are closely related, although not all Bayes factors are betting scores/ *e*-values. The bottom-line for communication purposes is that the reporting by ALL-IN meta-analysis can be interpreted in many ways— *p*-values, likelihood ratios, Bayes factors—but regardless of the interpretation provide fully frequentist type-I error control for tests and coverage for confidence sequences.

The remainder of this paper discusses the four categories of advantages in more detail: *Statistics* in Section 1, *Efficiency* in Section 2, *Collaboration* in Section 3 and *Communication* in Section 4. We use the Covid-19 vaccine trials as running examples, based on the FDA game described already, but also in terms of the *safe/e-value logrank test* ( Ter Schure *et al*., 2020b). We also briefly discuss an actual ALL-IN meta-analysis in Section 3.1, that used this safe/ *e*-value logrank test to study whether the Bacillus Calmette–Guérin (BCG) vaccine, originally developed to protect against tuberculosis and named after its inventors, could protect against Covid-19 ( Van Werkhoven *et al*., 2021, ALL-IN-META-BCG-CORONA). In the concluding section we will provide some broader context, with an overview of all the methods already developed— *e*-values, *safe* tests ( Grünwald *et al*., 2019) and *anytime-valid* confidence sequences— methods already available in software—notably safestats R package ( Turner *et al*., 2022)—and future work. R code for all calculations, simulations and plots is available in the software availability statement ( Ter Schure, 2021, https://doi.org/10.17605/OSF.IO/U6WTP). 

## 1 Statistics

The language of betting comes with the intuition that winning a large betting score has a small probability if the null hypothesis is generating our observations (e.g. the roulette wheel is fair). We will make this intuition precise and show how to control the type-I error by bounding this probability by Markov’s inequality and Ville’s inequality. Crucial here is that the betting score underlying our test is an *e-value*. The language of betting also comes with the intuition that when playing a game that is favorable to us in principle, we can use strategies of different quality: even among all strategies under which we expect to get richer, some of them can be expected to earn us much more than others. We will relate the more well known notion of power to such a different notion of *optimality*. In the following we discuss both *e-values* and *optimality* first for a single trial (in the FDA game and more generally) and then for ALL-IN meta-analysis. We conclude by a generalization of optimal *e*-value tests to confidence sequences.

### 1.1 Under the null: e-values in a single trial

To make the FDA game fair we imposed a multiplication by 2.4 (or 170/70) if we observe the event in the vaccine group and 1.7 (or 170/100) if we observe it in the placebo group. This multiplication has expectation 1 (or smaller) if we assume the null hypothesis of a vaccine with negligible VE of 30% (or smaller). In case of 30%, we have probability 0.41 (or 70/170) to observe a vaccine event and probability 0.59 (or 100/170) to observe placebo, so in expectation we multiply our investment by: 70/170 · 170/70 +100/170 ·170/100 = 1. No matter how we invest in the two outcomes, (e.g. putting 1/3 on vaccine and 2/3 on placebo, or something different) in expectation under the null we multiply the initial investment by 1. This means that our betting score is an *e*-value, since by definition an *e*-value is the outcome of a nonnegative random variable with expectation (at most) 1 under the null hypothesis ( Grünwald, 2021).

Our betting score could also be rewritten as a likelihood ratio, so the expectation of the likelihood ratio ((50% VE | *X*)/(30% VE | *X*)) is 1 as well. We hence-forth write the likelihood ratio after *n* rounds of betting (or after observing *n* events) as **LR** ^( *n*)^, with for the FDA game


$$\;LR^{\left(n\right)}=\prod_{i=1}^{n}\frac{L(50\%\;\text{VE}|X_{i})}{L(30\%\;\text{VE}|X_{i})}.\;(1)$$

Using its expectation of 1, Markov’s inequality bounds the probability of observing a large multiplication of our investment € (a large likelihood ratio) by *α* after *n* = 170 rounds as follows:


$$P_{\text{30\%}\;\text{VE}}\;\left[LR^{\left(170\right)}\geq 1/\alpha \right]\leq \frac{E_{30\%\;\text{VE}}\left[LR^{\left(170\right)}\right]}{1/\alpha }=\frac{1}{1/\alpha }=\alpha .$$

**Figure 1.**
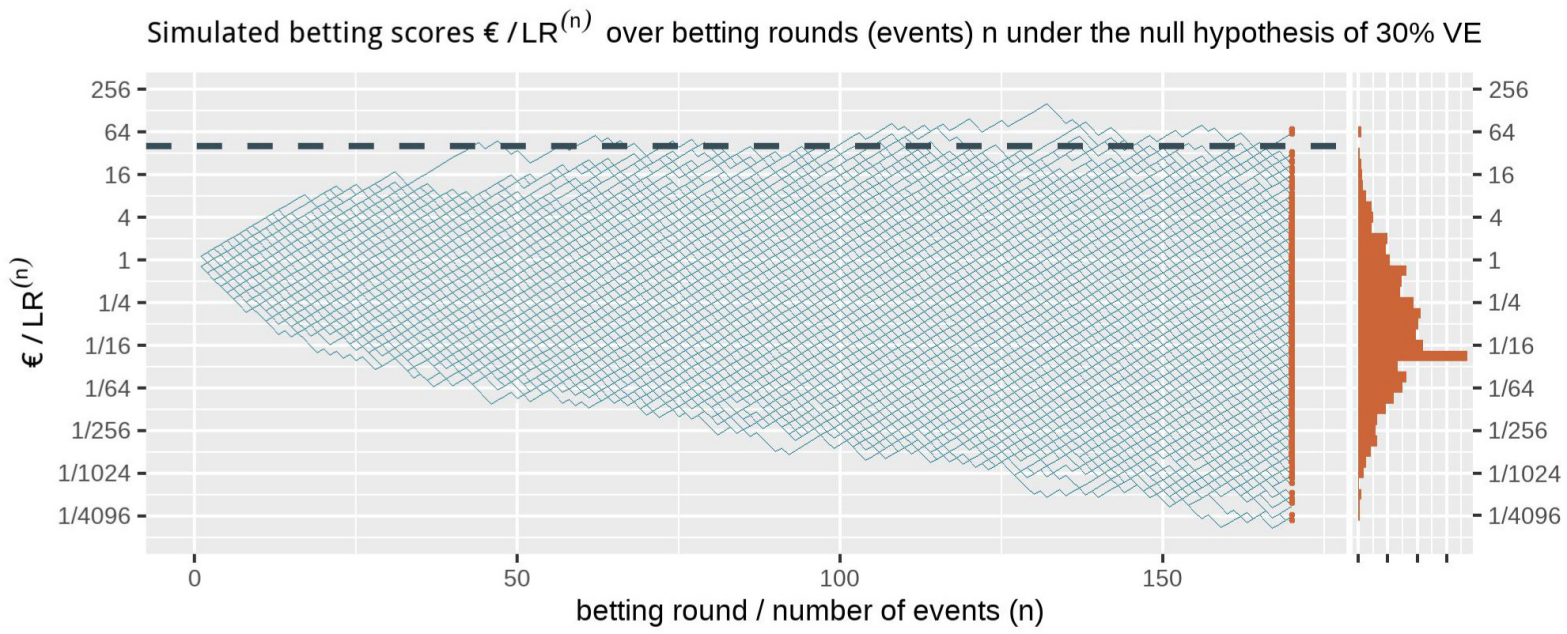
1000 simulated betting scores in the FDA game over betting rounds *n* assuming a probability of 0.41 (70/170) for each event to occur in the vaccine group (the null hypothesis of 30% VE). The dashed line is the threshold 1/ *α* = 40 one-sided. The histogram at the right shows the betting score/ *LR* ^(170)^ after 170 events. Note that the expectation of 1 of the scores is not the mode of its distribution nor its median and that the y-axis is on a log scale.

Figure 1 shows at the right side the histogram of betting scores in the FDA game after 170 events when we simulate events under the null hypothesis, with probability 0.41 to occur in the vaccine group, corresponding to 30% VE. A line is shown at 40, and indeed no more than *α* = 1/40 = 2.5% of the scores seem to be larger than that threshold. In fact, in these 1000 runs of simulation only 0.3% of the runs have a betting score larger than 40; Markov’s inequality is a loose bound. We also have a stronger result because we obtained our betting score over events by multiplying the score of the rounds (see ( 1), corresponding to reinvesting our winnings), called Ville’s inequality. We get the following from Ville (1939):


$$P_{\text{30\%}\;\text{VE}}\;\left[LR^{\left(n\right)}\geq 1/\alpha \;\text{for}\;\text{some}\;n\right]\leq \alpha .$$

Ville’s inequality is also illustrated in Figure 1: if we take the sequence of rounds into account, still only a few out of the 1000 simulations *ever* reach a betting score larger than 40. In fact, in these 1000 runs of simulation only 1.1% of the runs have a betting score that is larger at any round in the game, such that our type-I error is controlled at *α* = 2.5% at any time. Moreover, this type-I error control is not tied to this maximum number of 170 events, but continues to hold with an unlimited horizon. Making a large profit in such a fair game casts doubt on the null hypothesis and is captured by a likelihood ratio that grows away from 1: a large betting profit is obtained if the null likelihood is performing worse than alternative.

**When trials can be summarized as bets** Before they can be combined in a meta-analysis, individual trials are often characterized by the summary statistics from trial publications. Conventional meta-analysis combines these statistics (e.g. mean differences and standard deviations) in a *Z*-statistic ( Borenstein *et al*., 2009). Unlike the vaccine/placebo outcomes that we have seen so far, such a *Z*-statistic has a continuous density and cannot be summarized by separately dealing with all possible outcomes. Fortunately, Shafer (2021) shows that any likelihood ratio of distributions can be viewed as a betting score in a game with initial investment €1. This is possible because likelihood ratios have expectation 1 in general if we assume the null hypothesis in the denominator of the ratio. For a *Z*-statistic we have two normal distributions with variance 1, one with mean *µ*_0_ under the null hypothesis, and one with *µ*_1_ under the alternative. If the data is generated by the null model, the expectation of the likelihood ratio is


$$E_{Z\sim \phi_{\mu_{0}}}\left[\frac{\phi_{\mu_{1}}(Z)}{\phi_{\mu_{0}}(Z)}\right]=\int_{z}\phi_{\mu_{0}}(z)\frac{\phi_{\mu_{1}}(z)}{\phi_{\mu_{0}}(z)}\text{d}z=\int_{z}\phi_{\mu_{1}}(z)\text{d}z=1,\;(2)$$

since *ϕ*_*µ*_1__( *z*) is a probability density that integrates to 1. This means that any such likelihood ratio for a *Z*-statistic is an *e*-value and can be used to construct tests by betting.

Not all summary statistics can be assumed to form a *Z*-statistic with a normal distribution. Fortunately for the logrank statistic this is reasonable ( Ter Schure *et al*., 2020b) if studies are large and the effect size not too extreme (hazard ratios not too far away from 1). We will use the logrank *Z*-statistic as a running example for meta-analysis on summary statistics. For an IPD meta-analysis (on individual patient data), however, we recommend to use the exact safe/ *e*-value logrank test from Ter Schure *et al*. (2020b) that is valid regardless of the randomization (e.g. 1:1 balanced or 1:2 unbalanced), the number of participants at risk, the number of events or the size of the effect—so also for a hazard ratio 0.05 that corresponds to a VE of 95%.

### 1.2 Under the null: e-values in a (live) meta-analysis

Assume we want to perform a meta-analysis and we collect a *Z*-statistic *Z _i_* from each trial *i*, e.g. a logrank statistic. Before observing *Z _i_* we construct an honest bet **LR**_*i*_ = *ϕ*_*µ*_1__( *Z _i_*)/ *ϕ*_*µ*_0__( *Z _i_*) for each trial that is an *e*-value and thus has type-I error control under the null hypothesis *ϕ*_*µ*_0__—for a default logrank statistic this is always *µ*_0_ = 0 corresponding to hazard ratio of 1. If we think of the betting score from the first study and invest it in the second study, we are in fact multiplying likelihood ratios. We need to have a notion of time *t*, such that at each time we know the number of studies *k*〈 *t*〉 so far and the number of observations *n _i_* 〈 *t*〉 in each study *i*. If we assume that all studies are completed at time *t* with *n*_1_, *n*_2_,..., *n _i_* events summarized by logrank *Z*-statistics $z_{1}^{\left(n_{1}\right)},z_{2}^{\left(n_{2}\right)},\ldots ,z_{k}^{\left(n_{k}\right)}$ we can construct our ALL-IN bet as follows:


$$LR_{\text{META}}^{\text{〈}t\text{〉}}=\prod_{i=1}^{k\text{〈}t\text{〉}}LR_{i}^{\left(n_{i}\right)}=\prod_{i=1}^{k\text{〈}t\text{〉}}\frac{\phi_{\mu_{1}\sqrt{n_{i}}}(z_{i}^{\left(n_{i}\right)})}{\phi_{0}(z_{i}^{\left(n_{i}\right)})}.\;(3)$$

**The global null hypothesis** Each trial bet is testing the same null hypothesis *µ*_0_ = 0 in ( 3), such that the ALL-IN meta-analysis bet tests a *global null hypothesis* of no effect (0% VE) in all trials. Such a global null hypothesis can be rejected with a contribution from each trial, but also in case only one trial observes a large score betting against the hypothesis and no other trial observes a very small betting score that loses those winnings again. After all, the null in each trial is rejected as soon as the null is rejected in one of the trials.

**Meta-analysis on interim data** We can generalize this ALL-IN meta-analysis bet of completed trials to bets on interim data by assuming that we only have an interim logrank *Z*-statistic *z*_1_〈 *t*〉, *z*_2_〈 *t*〉,..., *z _k_* 〈 *t*〉 for the *n*_1_〈 *t*〉, *n*_2_〈 *t*〉,..., *n _k_* 〈 *t*〉 events observed so far at time *t*; *k*〈 *t*〉 still represents the number of studies so far at time *t*, but now these studies are not (all) completed. We construct our ALL-IN bet in a similar way:


$$\;LR_{\text{META}}^{\text{〈}t\text{〉}}=\prod_{i=1}^{k\text{〈}t\text{〉}}LR_{i}^{\left(n_{i}\text{〈}t\text{〉}\right)}=\prod_{i=1}^{k\text{〈}t\text{〉}}\frac{\phi_{\mu_{1}\sqrt{n_{i}\text{〈}t\text{〉}}}(z_{i}\text{〈}t\text{〉})}{\phi_{0}(z_{i}\text{〈}t\text{〉})}.\;(4)$$

From the perspective of Ville’s inequality, the analysis on completed trials and the one on interim data are indistinguishable. The only thing that matters is that we include all the data we have so far at time *t*, such that we have type-I error control


$$\;P_{0}\left[LR_{\text{META}}^{\text{〈}t\text{〉}}\geq 1/\alpha \;\text{for}\;\text{some}\;t\right]\leq \alpha ,\;(5)$$

for the global null hypothesis probability **P**_0_ with an unlimited horizon over time *t*.

### 1.3 Under the alternative: optimality in a single trial

A power analysis sets a very specific goal for a trial, usually to detect an effect of minimal clinical relevance. This is the effect we would not like to miss if it were there, although we hope that the real effect is larger. We nevertheless use this smallest effect of interest to decide on the sample size of the trial, otherwise we risk a futile trial. The FDA was clear on what this minimal effect should be for the Covid-19 vaccine trials: a VE of 50% ( FDA, 2020). This is the effect we used to bet in the FDA game.

**Figure 2.**
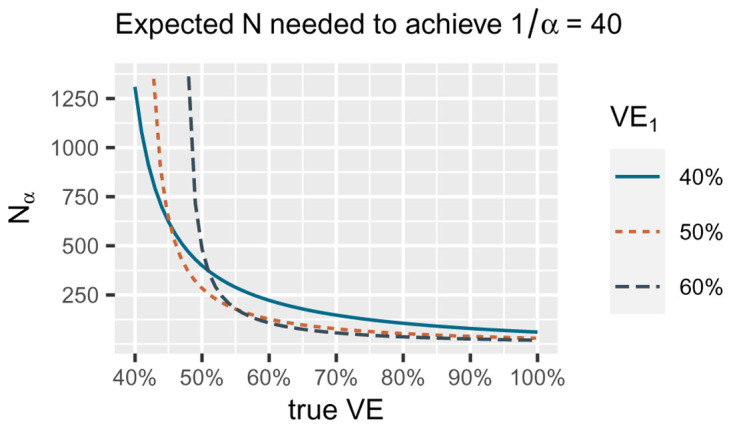
*N _α_* is the expected number of events needed to reach a betting score of 1/ *α* = 40 for *α* = 0.025 if we bet according to VE _1_ indicated by the three different lines, with bets each of the form $\prod_{i=1}^{N}\;\frac{L({\text{VE}}_{\text{1}}|X_{i})}{L(30\%\text{VE}|X_{i})}.$ The number of events we need decreases if the true VE underlying the data increases (the true difference in risk between vaccine and control is larger). The smallest number of events for a true VE of 40% is reached by betting VE _1_ of 40% (blue solid line), the smallest number of events for a true vaccine efficacy (VE) of 50% by betting VE _1_ of 50% (orange dotted line) and the smallest number for true VE of 60% by betting VE _1_ of 60% (grey dashed line). Note that for the alternative in the FDA game *H*_1_ = { *P*_VE_ : 50% ≤ VE ≤ 100%} we are only interested in playing the game well if the true VE is 50% or larger. Since for larger true VE, taking VE _1_ = 50% performs quite well, our strategy is to optimize for the worst case of 50% VE itself and use the bet with VE _1_ = 50% in the FDA game.

**Figure 3.**
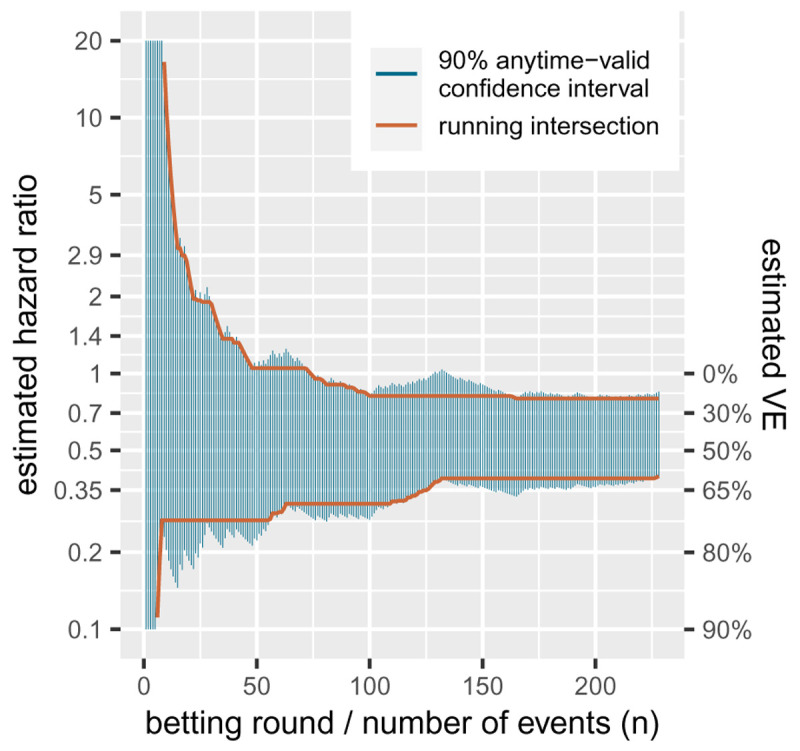
90%-confidence sequence for a random ordering of the 83 events in the vaccine group and 145 events in placebo from the CureVac AG (2021) trial. Note that the y-axis is on the log scale.

Our strategy in the FDA game, however, was not trying to achieve optimal power. If we compare the *all-or-nothing* confidence interval for CureVac AG (2021) from the introduction—the final analysis on 83+145 events—we notice that this confidence interval [25.3%, 57.1% VE] is smaller than the final anytime valid interval we show in Figure 3 in Section 1.5, which is [20.2%, 60.3% VE]. Note that we are comparing a *Z*_*α*/ _2__-confidence interval for *α*/2 = 0.02281 with *α*/2 = 0.05, so the wider interval cannot be attributed to the level of *α*. The difference is that the former one is optimized to have spent all *α* at the final analysis, while the latter one is optimized to continue data collection. Power is the probability of finding the desired result using the specified analysis at a sample size or stopping rule. So for an analysis that is intended to have unlimited horizon, power is not a well-defined concept. Instead Grünwald *et al*. (2019) introduced the concept of *growth-rate optimality in the worst case*, or *GROW*. Here, the goal is to optimize the expected rate at which the evidence grows (or the interval shrinks) for each new data point, not at a specific sample size. The worst case here is the 50% VE for a one-sided alternative hypothesis *H*_1_ = { *P*_VE_ : 50% ≤ VE ≤ 100%}. We optimized the FDA bet in the introduction by putting this 50% VE in the alternative likelihood. This can be rewritten in terms of a likelihood ratio for the logrank statistic *Z* as follows:


$$\begin{array}{l} LR^{\left(n\right)}=\prod_{i=1}^{n}\frac{L(50\%\;\text{VE}|X_{i})}{L(30\%\;\text{VE}|X_{i})}=\frac{L(50\%\;\text{VE}|X_{1},\ldots ,X_{n})}{L(30\%\;\text{VE}|X_{1},\ldots ,X_{n})}\\ \;\approx \frac{\phi_{\mu_{\min}\sqrt{n}}(Z^{\left(n\right)})}{\phi_{\mu_{0}\sqrt{n}}(Z^{\left(n\right)})}, \end{array}\;(6)$$

with *µ*_min_ = log(0.5)/4 and *µ*_0_ = log(0.7)/4 with 0.5 and 0.7 the hazard ratios corresponding to VE of 50% and 30% respectively (see Ter Schure *et al*. (2020b)). So our one-sided alternative hypothesis for the logrank *Z*-statistic is a *Z*-distribution with a mean representing an effect that is at least *µ*_min_:


$$H_{1}=\{\phi_{\mu_{1}}:\;\mu_{1}\leq \mu_{\min}\}$$

(since positive VE corresponds to a negative *µ*_1_). Our choice of the parameter of the alternative likelihood *µ*_min_ follows directly from the minimal effect set by the FDA. Kelly (1956) already showed that this way of betting optimizes the way our betting score grows if the true VE is 50% (our worst-case scenario). Breiman (1961) showed that this approach also minimizes the number of events we need to reach a given betting score set in advance (e.g. €1/ *α*), for which some intuition is given in Figure 2. Grünwald *et al*. (2019), Shafer (2021) and the appendix to Ter Schure *et al*. (2020b) give various other reasons why this is the best way to bet, relating it to data compression, information theory, Neyman-Pearson testing, Gibb’s inequality, and Wald’s identity. The most crucial property for the purposes of ALL-IN meta-analysis is that the alternative likelihood puts some money on each possible outcome, such that no matter what outcome we observe, we keep some of the money we risk. This contrasts the approach with a classic *p* < *α* test that essentially puts all money on the rejection region, such that if the outcome is not in it, we we lose all and cannot continue betting. A thorough interpretation of Neyman-Pearson testing and *p*-values in terms of betting is given by both Grünwald *et al*. (2019) and Shafer (2021).

### 1.4 Under the alternative: optimality in a meta-analysis

ALL-IN meta-analysis allows for a retrospective meta-analysis that is bottom-up. The betting score that we accumulate by reinvesting from one trial into the other (which is multiplying betting scores) has an interpretation without enforcing a common design or stopping rule on all included trials. This is especially important if trials have their own stopping rules, or if meta-accumulation processes are at play that influence the existence of trials based on earlier (trial) results in the same meta-analysis. While a meta-analysis can be bottom-up and each have its own design and effect of minimal interest, it can be advisable to agree on a *µ*_min_ for the meta-analysis. However, the meta-analysis betting score can also allow each trial *i* to have its own alternative likelihood with parameter *µ*_min( *i*)_. Then the following multiplication of those betting scores is still a valid meta score with type-I guarantees:


$$\;LR_{\text{META}}^{\text{〈}t\text{〉}}=\prod_{i=1}^{k\text{〈}t\text{〉}}\frac{\phi_{\mu_{\min(i)}\sqrt{n_{i}}}(z_{i}^{\left(n_{i}\right)})}{\phi_{0}(z_{i}^{\left(n_{i}\right)})}.\;(7)$$

As long as $\phi_{\mu_{\min(i)}\sqrt{n_{i}}}$ is a probability density that integrates to 1, we have that each likelihood ratio integrates to 1 under the global null hypothesis, such that ( 5) holds. This means that trials can also learn their parameter *µ*_min( *i*)_ from already completed trials. This is sometimes the case if trials are not powered to detect an effect of minimal interest, but an effect that is plausibly true based on earlier research. Kulinskaya *et al*. (2016) shows that such use of existing studies to power new trials can actually bias conventional meta-analysis since it introduces yet another dependency between sample size and results that is unaccounted for in any analysis that assumes a fixed sample size. For ALL-IN meta-analysis this is no problem at all, and trials can learn from each other as long as the parameter *µ*_min( *i*)_ is fixed before seeing new data that is evaluated using that parameter in ( 7). In Ter Schure *et al*. (2020b) we discuss the advantages of even learning the parameter within one trial using prequential plugins or Bayesian posteriors. In a game like the FDA game with a clear goal, this is inferior to the GROW approach, but in other situations it could be preferred. 

### 1.5 Confidence sequences

The CureVac AG (2021) trial reached their final interim analysis but was not able to reject the null hypothesis of 30%VE. The trial had been too optimistic and powered for 60% instead of 50% VE ( CureVac AG, 2020). If a trial is underpowered but still has a large number of participants in follow-up, there is good reason to continue the trial, or combine the trial with results from a new trial in a meta-analysis. However, with a total of 227 events this trial was not underpowered to reject the null hypothesis with an effect in the same ballpark as the Pfizer/BioNTech trial that reported 95% VE. In such a case it is very interesting to zoom in on the estimate for the effect, instead of its test.

A standard confidence interval can be seen as an inversion of a hypothesis test: if the null falls outside a two-sided 90%-confidence interval it can be rejected with a one-sided type-I error level of *α/*2 = 0.05. In general, the interval excludes all the values for the parameter that can be rejected. Similarly, in our context, an anytime-valid confidence interval excludes all values of the parameter that can be rejected by the *e*-value test that corresponds to the betting strategy at hand. So the interval is essentially tracking a whole range of bets, each against a different null hypothesis. Figure 3 gives a sequence of anytime-valid confidence intervals for a random ordering of the CureVac AG (2021) data, one for each new observed event or betting round. It shows that the more events we observe, the more parameters (hazard ratios, or their corresponding VEs) we can exclude from the interval. Because these intervals are valid at any time, once we can exclude a value, we never have to include it again. So we also show a sequence of intervals that is the running intersection of all the previous intervals. This of course crucially depends on the ordering, so the one shown for the CureVac AG (2021) data is just an example, since the ordering is not real. Since these intervals are anytime valid, it is possible to further shrink the intervals by continuing follow-up and observing more events. The coverage of an anytime-valid confidence sequence—like an *e*-value test—has an unlimited horizon.

An ALL-IN meta-analysis confidence interval that is based on a running intersection is of course only possible in an IPD meta-analysis, and cannot be based on summary statistics. The confidence interval shown in Figure 3 is based on the logrank *Z*-statistic (by repeatedly calculating it after each event), which can also be a summary statistic to achieve a single interval that is anytime-valid. The interval follows from the likelihood ratio of normal densities from ( 6) and follows a general recipe for constructing confidence sequences from Howard *et al.* (2021) where the hazard ratio is obtained by means of the Peto estimator ( Peto, 1987). The same approach can be used to obtain an ALL-IN meta-analysis confidence interval. A fixed-effects meta-analysis *Z*-statistic corresponds to a logrank statistic stratified by trial, and an estimate can be obtained from such a logrank statistic that Peto (1987) calls a *typical* hazard ratio. We discuss this approach a bit further in the final section.

## 2 Efficiency

Trials often suffer from recruitment difficulties, with estimates of 35% (between 1994 and 2002) and 56% (between 2004 and 2016) not reaching the goal set in advance ( McDonald *et al.,* 2006; Walters *et al.,* 2017). These trials find themselves underpowered according to their own protocol: when they decide the stop the recruitment and obtain the final sample size for analysis, they have a high probability for their test statistic to fall outside the rejection region they set in advance. This is exactly the scenario where meta-analysis could rescue the line of research by combining multiple underpowered trials. However, the literature on *research waste* ( Chalmers & Glasziou, 2009) and *Evidence-Based Research* ( Lund *et al.,* 2016) shows that we are not using the existing evidence base well to design the new trials needed for conclusion or to interpret new research. ALL-IN meta-analysis makes this very easy to do. It comes with a simple notion of the evidence already collected and what is still needed, and a notion of a new trial’s ability to provide that: the implied target. The combination of the two has the capacity to make study design more honest, showing what a trial can add to the existing evidence base instead of just evaluating a misguided goal to single-handedly answer a research question.

### 2.1 The evidence so far and what is still needed

An ALL-IN meta-analysis can set a prospective goal for conclusion, e.g. *α* = 0.0025 = 0.05 ^2 ^corresponding to the level of *α* required by authorities like the FDA that ask for two trials at the *α* = 0.05 level. Following Ville’s inequality ( 5) we need a betting score of 1/ *α* = €400 if we start with €1 to reach a conclusion. Because an ALL-IN meta-analysis combines trials by reinvesting or multiplying betting scores, a very simple calculation gives the betting score we still need at any given point. If an initial trial is able to reach a score of €8, any new trial can be designed to multiply that by 50. So on its own, starting with €1 instead of €8, it would need a betting score of €50 to help the meta-analysis reach €400. We could evaluate the sample size of the new trial on its ability to reach 50, which for a fixed sample size gives the conditional power of the meta-analysis once the new trial is added. However, if this second trial also foresees recruitment issues, it is more difficult to evaluate its planned contribution since it will probably not be the final trial in the meta-analysis. For this, Shafer (2021) proposed a new notion for the ability of a study, not to reach a specific target betting score, but as a continuous notion of how profitable it can be: the *implied target*. 

### 2.2 The ability of a new trial: the implied target

The likelihood ratio summarizes the data not in just two categories—statistical significant or not statistical significant—but captures the evidence so far on its way to a certain threshold. Similarly we propose to not evaluate experimental design as all-or-nothing, but summarize its ability to build on what is already there and facilitate future research. To capture a study’s expected contribution to a series of studies, we formulate the *implied target* from Shafer (2021) as the multiplicative amount with which the combined evidence is expected to grow if the study—designed with a certain *µ*_min _and sample size *n*—is added. In general, the implied target *E* ^*^ is defined as:


$$E^{*}=\exp\left(E_{Z^{\left(n\right)}\sim \phi_{\mu_{\min}\sqrt{n}}}\left[\log\left(LR^{\left(n\right)}(Z^{\left(n\right)})\right)\right]\right).\;(8)$$

**Figure 4.**
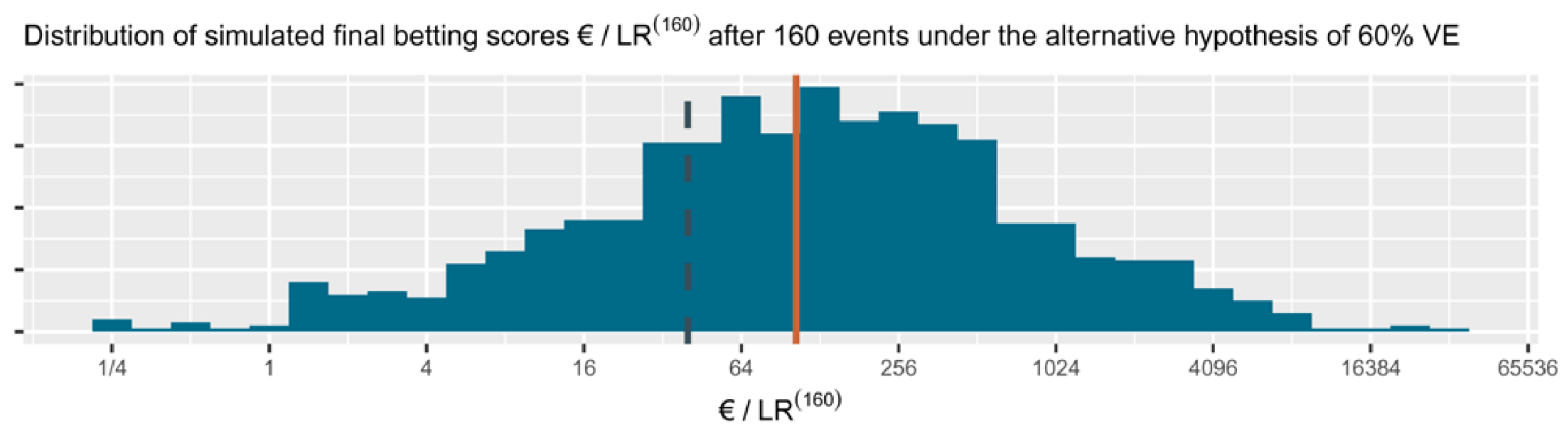
(and Figure 5) 1000 simulated sequences of betting scores by round in the FDA game after 160 events assuming a probability of 0.29 (40 */*140) for each event to occur in the vaccine group. This is the alternative hypothesis of 60% vaccine efficacy (VE) used to power the CureVac AG (2020) trial at a number of events of 160. The dashed line is the threshold 1 */α* = 40 one-sided and the solid line is the implied target of €104. Note that the x-axis is on a log scale.

The logarithm appears in equation ( 8) because the distribution of a likelihood ratio based on *n* events is very non-symmetric and heavy tailed, with extremely large likelihood ratios occurring with not so small probability (see Figure 4). So the expectation of the likelihood ratio is drawn very far from its typical values by these large likelihood ratios and is not a good expression of what to expect. The logarithm makes the distribution more symmetric (asymptotically (for large *n*) and for normal likelihood ratios even normally distributed), such that the expectation is a more meaningful summary of the evidence promised by the study. By exponentiation (exp()) we bring this expectation back to the scale of the likelihood ratio, such that it can be interpreted as a betting score or *e*-value.

**Figure 5.**
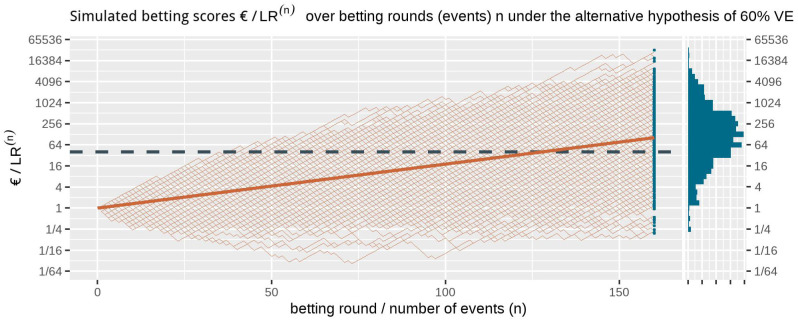
(See above at Figure 4.) The histogram for the final betting scores at the right shows the larger scores above and the smaller ones at the bottom, which means that if we turn it, it is the mirror image of the histogram in Figure 4. The dashed line is the threshold 1 */α* = 40 one-sided. The increase in the solid line per additional event/betting round shows the contribution to the implied target of each event, up until the implied target at *n* = 160 of 104. In this figure, the design has an approximate 79% power to observe a betting score/ *e*-value larger than 1 */α* = 40 before 160 events and 72% power at exactly 160 events (better visible in Figure 4). Note that the y-axis is on a log scale.

In the FDA game the expected growth rate per new event in the CureVac trial, assuming their effect of minimal interest of 60% VE, is the following:


$$\begin{array}{l} \exp\left(E_{60\%\;\text{VE}}\left[\log\left(\frac{L(50\%\;\text{VE}|X)}{L(30\%\;\text{VE}|X)}\right)\right]\right)\\ \;=\exp\left(\frac{40}{140}\text{⋅}\log\left(\frac{50/150}{70/170}\right)+\frac{100}{140}\text{⋅}\log\left(\frac{100/150}{100/170}\right)\right)\\ \;=1.029454. \end{array}$$

The cumulative contribution of each new event is shown as the linear line on the log scale in Figure 5. The CureVac AG (2020, Table 8) design planned a final analysis at *n* = 160 events, so their implied target was 1.029454 ^160 ^≈ 104. In comparison to the target score of €104 at 160 events, the actual betting score €1.84 after 83 + 145 = 228 events in the press release is quite disappointing. Shafer (2021) gives more examples of how betting scores and implied target help to interpret study results in the context of study design. 

### 2.3 Honest study design

An implied target does require an honest proposal of the effect of minimal clinical interest *µ*_min_, to evaluate the merits of the study. In regular power analysis, this parameter might be tweaked—e.g. setting an unrealistically large effect—to still argue for the study’s advancement with only small sample size. Or the smallest effect size of interest analysis is set after data is observed ( Wang *et al.,* 2018). This behavior is incentivized by the *all-or-nothing* character of Neyman-Pearson tests that also make the power analysis all-or-nothing. If your desired sample size does not meet the power hoped-for, you need to either increase it or abandon the study. This aspect of traditional analyses fully ignores the ideal of cumulative science in which one study is not expected to single-handedly answer a research question and small increments in knowledge are valuable, as long as they build towards a common goal. If they use *e*-values and the ALL-IN framework, researchers do not have to view their analysis as the final one, which helps them to evaluate their study more honestly ( Lakens, 2021).

## 3. Collaboration

The *Evidence-Based Research Network* ( Lund *et al.,* 2016) aims to always inform new research by past results and to reduce research waste by separating research ideas that are necessary from those that are wasteful. This is not easy to do, however. Different communities might have different notions of necessity or even of what is ethical (a state of so-called *clinical equipoise* ( Shamy *et al.,* 2020)). It might therefore be very beneficial to have all those running new clinical trials in a field collaborate together in an ALL-IN meta-analysis.

### 3.1 ALL-IN-META-BCG-CORONA

We ran two ALL-IN meta-analyses during the Covid-19 pandemic with the involvement of seven trials in one and four in the other. All were designed to study whether the BCG vaccine, originally developed to protect against tuberculosis, could protect against Covid-19 (based on a theory of non-specific immune effects and innate immunity ( Netea *et al.,* 2020)). The two meta-analyses study different populations (healthcare workers and the elderly) and two questions each: the effect of the BCG vaccine on Covid-19 infection (not necessarily symptomatic) and the effect on severe Covid-19 (indicated by hospitalizations). In the following description we will focus on the analysis of Covid-19 infections in the healthcare workers population.

ALL-IN-META-BCG-CORONA followed many of the steps also outlined by Tierney *et al.* (2021), that we will briefly discuss here: (1) Meta-analysis design, (2) Systematic search for trials, (3) Systematic review for trial inclusion (4) Data upload, and (5) Disseminating results.

**(1) Meta-analysis design** Early in the project we decided to aim for an IPD meta-analysis on interim data and wrote our protocols and statistical analysis plans. This timestamped two important decisions on the meta-analysis design: the hazard ratio of minimal interest of 0.8 (20% VE) for events of Covid-19 and the level of *α* set at 0.0025 so the threshold for the *e*-value was at 1 */α* = 400. For these decisions we set up a meta-analysis Steering Committee that was still fully blinded to any results at the time. The design was preregistered ( Van Werkhoven *et al.,* 2021) and all documentation and a webinar explaining the methodology were made available on a project website ( Ter Schure *et al.,* 2020a).

**(2) Systematic search for trials** We continuously searched for trials to include in the meta-analysis. Some were already known to our Steering committee before we started. They initiated a BCG trial of their own very early in the pandemic and shared their protocol with many of their contacts in the BCG research community. Other trials were found by a repeated systematic search of trial registries. The trials that agreed to join the meta-analysis were each represented by a member in the Advisory Committee. Meetings of the Advisory committee were scheduled regularly and the trials involved could point us to any new developments. A major advantage of ALL-IN meta-analysis here is that the number of trials does not need to be specified in advance.

**(3) Systematic review for trial inclusion** We received external advice from Cochrane Netherlands on trial inclusion based on a thorough risk-of-bias assessment. For this assessment, each trial shared their protocols, and subsequently all Cochrane’s evaluations were shared and discussed with the Steering committee and Advisory committee (where trials were usually represented by their PI’s who were blinded to any trial results). Trials had multiple opportunities to answer questions—from Cochrane Netherlands as well as other trials involved—explain their trial and express other concerns about differences between the trials included. The Steering Committee made the final decision on including a trial, before any of that trial’s results were known to anyone part of the discussion. The decision of the Steering committee explicitly incorporated both trial quality and meta-analysis homogeneity.

**(4) Data upload** Parallel to the discussions on trial inclusion, data transfer agreements were signed and data was shared through a secure upload. Each trial had a data uploader that was in close contact with the ALL-IN metatrial statistician (the first author of this paper) about data quality. The ALL-IN statistician did not attend the discussion meetings and kept the Steering committee and Advisory committee blinded to any results before each trial inclusion decision.

**Figure 6.**
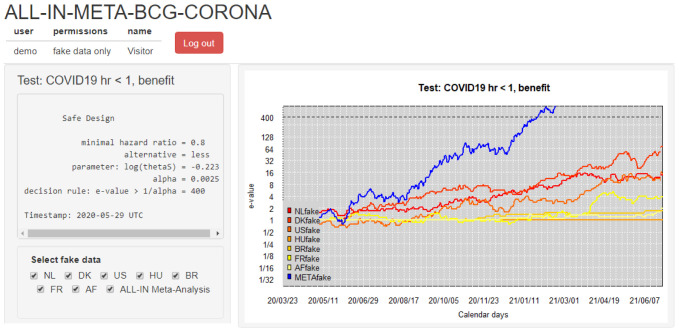
Dashboard used to communicate interim results in *ALL-IN-META-BCG-CORONA* to all data uploaders with a login. The involved trials were performed in the Netherlands (NL), Denmark (DK), the United States (US), Hungary (HU), Brazil (BR), France (FR) and Guinea-Bissau/Mozambique (AF). The dashboard is in demo mode and shows synthetic (“fake”) data. The option to (de)select trials is for plotting purposes of individual trial *e*-values; all trials in the dashboard stay included in the meta *e*-value, following the decision from the Steering committee on trial inclusion. Note that the y-axis is on the log scale.

**(5) Disseminating results** Each data-uploader received a dashboard account with permissions to inspect the meta-analysis *e*-value and their own trial contribution. Their access of interim meta-analysis results in the dashboard served as a motivator to keep their own trial data upload up-to-date and to check the sequence of *e*-values for errors. Figure 6 shows this dashboard based on a demo login with synthetic data (this demo was available for everyone involved to get an impression). After an initial period where the data-uploaders could only inspect their own trial results, they granted each other permission to inspect all the individual trial contributions. When the first trials were completed and the meta-analysis was approaching its conclusion, the results were also presented to the Advisory and Steering committees. Any interim decisions were planned based on the ALL-IN meta-analysis *e*-values, but results were also presented in the context of power, implied target per trial and confidence sequences. 

### 3.2 Collaborative and bottom-up meta-analysis

In many aspects, our approach agrees with the framework for prospective, adaptive meta-analysis ( *FAME*) proposal from Tierney *et al.* (2021). FAME argues for prospective meta-analyses in close collaboration with ongoing trials to achieve the same advantages outlined in this paper, such as aligning trial characteristics (“minimize heterogeneity”) and reducing publication bias and “bias [in] both review and meta-analysis methods” introduced by “prior knowledge of trial results” (e.g. accumulation bias ( Ter Schure & Grünwald, 2019)). In alignment with the FAME recommendations, ALL-IN-META-BCG-CORONA was prospectively designed by preregistering an overall effect size of minimal interest and an *α*-level. However, ALL-IN meta-analysis in general also allows for a more bottom-up approach when each trial’s *e*-value is based on that trial’s own design (effect size of minimal interest, see Section 1.4) and trial evidence is synthesized more loosely without a strict decision rule. In comparison to FAME, ALL-IN meta-analysis is much more adaptive. FAME proposes to use conventional meta-analysis (with a fixed sample size) and optimize the timing of the meta-analysis “to anticipate the earliest opportunity for a potentially definitive meta-analysis”. In that sense, FAME can only adapt to the speed of recruitment, while ALL-IN allows to adapt to any information so far including the evidence in the trials and the synthesis of the meta-analysis itself. There is also a statistical inconsistency in the FAME approach that is concerned with “striking a balance between maximising the absolute and relative information size and producing a sufficiently timely review” but does explicitly state that the last step of the meta-analysis is to “assess the value of updating the systematic review and meta-analysis”. A fixed sample-size statistical analysis should not be reanalyzed using the same statistical methodology. Any proposal to use conventional meta-analysis for efficiency purposes risks accumulation bias ( Ter Schure & Grünwald, 2019) because the timing of the meta-analysis might be driven by some of the results part of that same analysis. Hence the best approach is to combine the recommendations from FAME ( Tierney *et al.,* 2021) with statistical approach from ALL-IN meta-analysis and the spirit of living systematic reviews.

**Collaboration using a dashboard** A dashboard for ALL-IN meta-analysis allows us to spot trends in the accumulating evidence, or allow other stakeholders to monitor. A dashboard like Figure 6 can give access to the accumulating *e*-values to those that need to prepare for crossing a threshold in the near future, e.g. for independent data monitoring committees of ongoing trials or for those considering new trials or preparing to update medical guidelines. On a log-scale, the increase in *e*-values is linear (in expectation) and the observed trends can be estimated, e.g. in Figure 6 as an increase in evidence per additional calendar day.

For ALL-IN-META-BCG-CORONA, the time unit *t* in the definition of **LR** ^〈 *t*〉^ from ( 4) was set to calendar days and the *e*-values were updated at each calendar day with an event. The dashboard plots in Figure 6 horizontal lines at 1 for trials that do not observe any events yet: they have not started betting and are still at their initial investment of €1 contributing a neutral amount to the multiplication meta- *e*-value. ALL-IN meta-analysis monitors *e*-values as events come in, also when they do so from multiple trials simultaneously. In the language of betting, even the analysis of simultaneous events is considered a sequential bet. If the bet on the events from one trial pays out €4, it multiplies our initial capital by 4, and if the events from another trial pay out €5, it does so by a factor 5. Yet if we actually consider those trials to be consecutive bets, we reinvest the €4 from the first into the second, and obtain €1 ⋅ 4 ⋅ 5 = €20, as follows from the definition of the meta-analysis *e*-value on interim data in ( 4).

Figure 6 illustrates what going ALL-IN means: the evidence in all studies can be monitored and compared to the required threshold at any time. The hypothesis test is carried out by comparing the meta *e*-value in blue to the threshold 1 */α* of 400, plotted as a dotted line. Because the meta-analysis is *anytime* and *live* a conclusion is reached whenever the *e*-value sequence passes that threshold. The synthesis of studies can efficiently lead the decision to stop recruiting, treat the placebo group or discourage new trials to start, while encouraging inspection of each individual trial’s contribution to the meta-analysis. Since each trial’s contribution is a simple multiplication, their components can often be conveniently spotted in the agreement of the shape of the meta-analysis and individual trial lines in a dashboard like Figure 6 (as long as not too many trials are contributing simultaneously).

**Collaboration in a competitive field or a pandemic** ALL-IN meta-analysis also prevents losing type-I error control when many trials compete for answers on the same research question, e.g. in an uncoordinated scientific response to a pandemic. If trials are only evaluated in isolation and a response follows the first positive result of a single trial, serious multiple testing issues arise that inflate the type-I error and result in unreliable inference and, subsequently, poor decisions. This happens especially if all trials perform interim analyses on their own, and a type-I error occurs at an interim analyses before any other trial results are published to refute it. The example dashboard also clearly demonstrates decreased type-II errors: synthesizing the evidence in a meta-analysis at interim stages of the trials, and not after trials are completed, improves the ability to find an effect early. Collaboration is indeed much more efficient. 

### 3.3 Fixed-effects and random-effects meta-analysis

Sutton *et al.* (2007, p. 2491) note that “in a meta-analysis with considerable heterogeneity, the impact of a new (large) study will be (much) less in a random compared to fixed effect model”. This is due the incorporation of a parameter in the model that represents the between-study variation. Also Kulinskaya & Wood (2014) find that the goal of sequentially updating a random-effect meta-analysis might involve planning a large number of small trials to estimate the between-study variance well. Even if that is considered advisable, a random-effects model result might still be very difficult to interpret ( Riley *et al.*, 2011). Hence there are various reasons to prefer the fixed-effects model to monitor evidence efficiently and to ensure that the trials are sufficiently homogeneous.

Alongside ALL-IN-META-BCG-CORONA we initiated a second ALL-IN meta-analysis. While the first included trials on healthcare workers, the second included trials in the elderly. Early in the process, before seeing any data, our Steering committee noticed that the two groups could be very different. Based on a theory of innate and trained immunity, they expected a different effect of the BCG vaccine on the younger immune system of healthcare workers than on the older immune system in the elderly. It could even be that the BCG vaccine effect was beneficial in the ability to fight off Covid-19 in one population but harmful in the other. In general, the differences between trials can be in three categories: heterogeneous effects, conflicting effect and multiple testing.

**Heterogeneous effects** Our Steering committee decided that to declare success, all included trials in health-care workers should observe an effect of 20% VE or larger. If they indeed do, heterogeneity in their effect sizes (e.g. one 20%, one 50%, one 25%) does not matter for their joint ability to reject the *global null hypothesis* of no effect in all trials. So for testing the global null, trials are allowed to be heterogeneous in where they are in the space of the alternative hypothesis *H*_1_ = {VE: 20% ≤ VE ≤ 100%}. For estimation, however, it is not clear what the ALL-IN confidence interval is estimating if we assume that the effects in the trials are very different. Still, as a first summary, a *typical effect size* ( Peto, 1987) might be useful if we are unable to estimate a random effects model. The development of confidence sequences for random-effects meta-analysis is a major goal for future work. We do not, however, believe that the evidence in a line of research should be monitored based on whether this interval excludes the null hypothesis, or whether the *e*-value corresponding to the random-effects null model does: for testing, the global null is much more natural. Waiting for a random-effect model to reach a certain threshold is counter-intuitive, since it might require many small trials to estimate the between-trial variability instead of focusing on testing the treatment effect. Moreover, the goal of rejecting the null hypothesis corresponding to this model can be quite strange. When testing a zero-effect null hypothesis, it assumes that there are true effects of harm and true effects of benefit among the trials and that their mean is exactly zero.

**Conflicting effects** If one of the trials has an effect smaller than 20% or even a harmful effect, we should anticipate betting scores or *e*-values that are smaller than 1. So a meta-analysis multiplication of those *e*-values would reduce the evidence available from other trials. If we can identify groups for which we expect that the trials in each group have an effect in the same direction and of at least the minimal size, we can perform separate meta-analyses. This was the rationale behind grouping healthcare workers and the elderly each in their own ALL-IN-META-BCG-CORONA analysis.

**Multiple testing** When our analysis is exploratory, and we really have no idea how to group the various trials, we are faced with a multiple testing problem. Note that in this situation also no conventional meta-analysis method would be used to test a common null-hypothesis. We wonder whether any of the trials has the ability to reject the null hypothesis. In that case, we can divide our initial investment over the trials, and see if the totality of their bet achieves a high betting score. Research into this use of *e*-values has shown that indeed averaging *e*-values is the optimal way to have type-I error control in a standard multiple testing setting ( Vovk & Wang, 2021). We return to the notion of hedging bets and averaging *e*-values in Section 4.

Problems with heterogeneity in meta-analysis are not tied to the ALL-IN approach and familiar to anyone working with meta-analysis methods. ALL-IN-META-BCG-CORONA had the advantage that many of the trials that started later had drawn inspiration from the protocol of the first trial. The same sort of alignment of inclusion criteria and outcome definitions might be achieved in other lines of research as well. Hence close collaboration can be very important and the promise of an early conclusion of the research effort might keep a research field motivated to keep the goals aligned.

## 4. Communication

We have illustrated that the language of betting can be useful in interpreting results from an ALL-IN meta-analysis. Here we argue this further by giving extensions of our method that are very easily explained in terms of betting.

### 4.1 The language of betting for two-sided tests

Our examples so far covered one-sided tests, but those can be easily extended to two-sided tests, e.g. by taking


$$\begin{array}{c} LR_{\text{two-sided}}^{\left(n\right)}=\frac{1}{2}\cdot \left(LR_{\text{left}}^{\left(n\right)}+LR_{\text{right}}^{\left(n\right)}\right),\\ \text{with}\\ LR_{\text{left}}^{\left(n\right)}=\frac{\phi_{\mu_{\min(\text{left})}\sqrt{n}}(z^{\left(n\right)})}{\phi_{\mu_{0}}(z^{\left(n\right)})}\;\text{and}\;LR_{\text{right}}^{\left(n\right)}=\frac{\phi_{\mu_{\min(\text{right})}\sqrt{n}}(z^{\left(n\right)})}{\phi_{\mu_{0}}(z^{\left(n\right)})}, \end{array}$$

to represent a two-sided alternative hypothesis


$$H_{1}=\left\{\phi_{\mu_{1}}:\;\mu_{1}\leq \mu_{\min(\text{left})}\;\text{or}\;\mu_{1}\geq \mu_{\min(\text{right})}\right\}.$$

Such a two-sided test is easy to interpret in the language of betting. We essentially split our initial investment (e.g. €1) between the two sides of the alternative hypothesis (e.g. by betting €0.50 on one side and €0.50 on the other). Any other weighting of the two sides is also possible and corresponds to a different division of the initial investment. The crucial thing is that each side tests the same null hypothesis *H*_0 _ = { *ϕ*_*µ*_0__} and has expectation 1 under the null hypothesis, such that any weighted average also has expectation 1 and is an *e*-value. Note that for a meta-analysis at time *t* with *k*〈 *t*〉 studies this becomes:


$$LR_{\text{two-sided}}^{\text{〈}t\text{〉}}\;:\text{=}\frac{1}{2}\left(\prod_{i=1}^{k\text{〈}t\text{〉}}LR_{i,\text{left}}^{\left(n_{i}\text{〈}t\text{〉}\right)}+\prod_{i=1}^{k\text{〈}t\text{〉}}LR_{i,\text{right}}^{\left(n_{i}\text{〈}t\text{〉}\right)}\right).\;(9)$$

Usually one side of the bet is losing and the other is winning such that we do not want to reinvest (multiply) across sides but keep them separate for all trials. In our ALL-IN-META-BCG-CORONA dashboard we also visualized these two sides of the meta-analysis test separately; in Figure 6 we show only the left-sided test (for benefit) of the two.

### 4.2 The language of betting for co-primary endpoints

Another way to hedge our bets is by considering multiple primary outcomes. In ALL-IN-META-BCG-CORONA, for example, not only were the Covid-19 events counted, but Covid-19 hospitalizations as well, as an indicator for severe disease. We started with *α* = 0.05 and put 10% on Covid-19 ( *α* = 0.0025 on each of the two sides of a two-sided test) and 90% on hospitalisations ( *α* = 0.0225 on each of the two sides of a two-sided test). So the thresholds to achieve with the *e*-value for Covid-19 was set at 1 */α* = 400 and the one for hospitalization at 1 */α* = 44.44. A different way to formulate this is that each had to achieve 1 */α* = 20, but that the sequence of *e*-values for Covid-19 started with an initial investment of €0.05 for each side of the two-sided test (and had to multiply by 400 to reach €20) and that the *e*-value for hospitalization started with an initial investment of €0.45 for each side (and had to multiply by 44.44 to reach €20).

There are two ways to consider such a bet on two co-primary outcomes: separately and combined. If we evaluate the *e*-values for each primary outcome separately and reach the threshold with either of the two, we are rejecting the null for that outcome. We are doing two separate tests. If we evaluate the *e*-values combined, we average them weighted by their *α*, just as for the two sides of the two-sided test. In that case we have similar type-I error control, but reject the null hypothesis that both are a null effects in favor of the alternative hypothesis that one of them is not. Yet we cannot conclude which one is non-null with the same type-I error since our *α* level applies to the combined bet and the individual components to the averaged bet are essentially lost. 

## Concluding remarks

The novelty of this paper lies in a new method for meta-analysis. We do not claim any novelty for the underlying mathematics, though. The basic methods we describe can be viewed as relatively minor variations of the anytime-valid tests that are designed to preserve type-I error under optional stopping, as designed by H. Robbins and his students ( Darling & Robbins, 1968; Robbins, 1970). Unfortunately and surprisingly, these tests have not caught on in statistics until a few years ago—right now they are thriving in work on so-called *safe tests*, *anytime-valid confidence sequences* and *e*-values e.g. Grünwald *et al.* (2019); Henzi & Ziegel (2021); Howard & Ramdas (2019); Howard *et al.* (2021); Johari *et al.* (2021); Pace & Salvan (2019); Ramdas *et al.* (2020); Shafer *et al.* (2011); Shafer (2021); Turner *et al.* (2021); Vovk & Wang (2021). As far as we know, it has never before been suggested to use such methods in a meta-analysis context. (Group sequential methods, which have originally also been inspired by the anytime-valid tests, have in turn spurred developments in meta-analysis, but these are substantially different from ALL-IN.) Also, the fact that the logrank test can give a likelihood ratio of the type needed for an anytime-valid test/an ALL-IN meta-analysis is a new finding described by Ter Schure *et al.* (2020b).

### Likelihood ratios, *E*-variables and *e*-values

In this paper we presented betting scores/ *e*-values that are equivalent to likelihood ratios. In general though, betting scores and *e*-values are really generalizations of likelihood ratios that preserve the properties of likelihood ratios that give them a prominent role in statistics. Entire books have been written to advocate for summarizing evidence in observed data by a likelihood ratio ( Edwards, 1974; Royall, 1997) and to separate the goal of measuring evidence from expressing posterior beliefs and making decisions. Likelihood ratios have the property that they can “favor a true hypothesis over a false one more and more strongly” and while a likelihood ratio can be misleading, “strong evidence cannot be misleading very often” ( Royall, 1997, p. 14). This latter type-I error control is also referred to as a *universal bound* by Royall (1997) and, by recognizing Ville’s inequality, can be generalized to other betting scores and *e*-values.

 A betting score € is a random outcome of a bet and its random variable is an *E*-variable if it is nonnegative and for all *P* ∈ *H*_0_, **E**_*P*_[€] ≤ 1. For a given outcome of the bet, the value of such a random variable is the *e*-value. Ville’s inequality relies on the multiplication of *E*-variables—forming a test martingale—which also has expectation smaller than 1 and thus is itself an *E*-variable. For the example *e*-values in this paper, the requirement on the expectation **E**_0_[ **LR**] ≤ 1 holds for a simple null hypothesis, e.g. *H*_0 _ = { *ϕ*_0_}.

 Apart from likelihood ratios of two simple hypotheses, *e*-values can also be defined for more complicated tests—e.g. a *t*-test with a nuisance parameter for the variance—in which case the unit expectation needs to hold not for a single mean-0-normal distribution with known variance, but for all mean-0-distributions with any variance. Grünwald *et al.* (2019) shows that it often is possible to construct *E*-variables for such composite testing problems, which is why we consider the *e*-value the right generalization of the likelihood ratio. 

### Anytime-valid confidence sequences

In this paper we briefly presented a confidence sequence (in Figure 3) for the hazard ratio or VE that was based on the Gaussian approximation to the logrank statistic and the Peto (1987) estimator. This estimator can be derived from summary statistics and is therefore still quite common in meta-analysis as a so-called *two-stage method*, although it is advised against for extreme hazard ratios ( Simmonds *et al.,* 2011). Research into other confidence sequences for the hazard ratio is still ongoing. For other estimation problems, confidence sequences already have been thoroughly studied, for example for medians and other quantiles ( Howard & Ramdas, 2019), and odds ratios ( Turner *et al.,* 2021). These have not, however, been extended to meta-analysis, and especially for the random-effects meta-analysis model, research into confidence sequences is a major goal of future work.

## Data availability

No data are associated with this article.

## Software availability

The safestats R package ( Turner *et al.,* 2022) provides software to do an *e*-value analysis for the *t*-test, *Z*-test, logrank test and 2 *x*2-tables. Also functions are available to calculate the power and implied target for these study designs. Confidence sequences can be calculated for the odds ratio in 2x2-tables and the hazard ratio in time-to-event data.

R code for the calculations, simulations and plots in this paper can be found on the Open Science Framework ( Ter Schure, 2021, https://doi.org/10.17605/OSF.IO/U6WTP).

These were produced using the GitHub version of the safestats R package accessed 22 September 2021 ( Ly *et al.,* 2021).

This code is available under the terms of the Creative Commons Zero "No rights reserved" data waiver (CC0 1.0 Public domain dedication). 

The code that produces the dashboard in Figure 6 is not publicly available since it mainly deals with logins and permissions that concern those involved in the particular ALL-IN meta-analysis that is described there as an example.
