# Supplementary material for: ALL-IN meta-analysis: breathing life into living systematic reviews and prospective meta-analyses
Source: F1000Res. 2025 Jun 19;11:549. Originally published 2022 May 19. [Version 2] doi: 10.12688/f1000research.74223.2 (PMC9587381; doi:10.12688/f1000research.74223.2)
Supplement: Peer review history

# Reviewer report: Reviewer response for version 2

**DOI:** 10.5256/f1000research.178216.r393268

**Published:** 2025-08-12

**Authors:** Shubhendu Trivedi

**Affiliations:** 1 Massachusetts Institute of Technology (MIT), Cambridge, MA, USA

No further comments to make. The revised version improves on the original manuscript, which was already top notch in my view.

Is the rationale for developing the new method (or application) clearly explained?

Yes

Is the description of the method technically sound?

Yes

Are the conclusions about the method and its performance adequately supported by the findings presented in the article?

Yes

If any results are presented, are all the source data underlying the results available to ensure full reproducibility?

No source data required

Are sufficient details provided to allow replication of the method development and its use by others?

Yes

Reviewer Expertise:

Machine Learning, Statistics, Conformal Prediction, Sequential Testing, Drug Discovery.

I confirm that I have read this submission and believe that I have an appropriate level of expertise to confirm that it is of an acceptable scientific standard.

# Reviewer report: Reviewer response for version 1

**DOI:** 10.5256/f1000research.77953.r146412

**Published:** 2022-10-21

**Authors:** Shubhendu Trivedi

**Affiliations:** 1 Massachusetts Institute of Technology (MIT), Cambridge, MA, USA

The paper presents a new method for meta-analysis, motivated partly by (and for "breathing life" into) living systematic reviews that are used in the clinical domain which provide recommendations to prevent research waste. The authors christen their method to be ALL-IN meta-analysis, which is Anytime Live and Learning Interim Meta-Analysis. "Anytime" meaning analysis can be updated at any time and can control for type I error irrespective of any other decision making along the way. "Live" allows for a bottomup collaboration of different trials; a trial can be initiated in any way, and we can include data from the meta-analysis itself. "Interim" permits for a combination of data from trials that are still ongoing. 

 The paper begins with a topical motivation from the covid19 pandemic, while emphasizing that the methodology could help better evidence combination, collaboration, and communication during later pandemics, or even smaller clinical trials. Using a single trial and specifications issues by the FDA for the covid19 vaccine trails (regarding vaccine efficiency and evidence against a null hypothesis say 30% VE) , the general betting game that is the language central to much of the contribution of the paper is introduced. It is shown that the same can also be written in terms of likelihood ratios and examples for scores are calculated for a Pfizer trial and a CuraVac trial. 

 The betting based methodology allows the statistical analysis to not simply be all or nothing (like p testing). In the all or nothing setting, we can not continue from one trial to another without violating type I error rates, while in the betting (ALL-IN) setting one can simply update patients later on. This also permits for better efficiency (we can understand the number of participants needed to answer a research question) and collaboration (since we can combine analysis as data becomes available). The language of betting also can be interpreted in various equivalent ways (likelihood ratios, conservative p values, e values) that also allow for easy and crisp communication about the analysis. 

 The intuitions of the language of betting are made more precise using standard tools in the literature (Markov's inequality, and Ville's inequality). Further, the betting score underlying the test is an e-value which further permits statistical analysis (using the tools cited). logrank Z statistics are used as a running example for meta-analysis on the summary statistics -- we can simply collect the Z statistics Z_i from each trial, which can easily be combined (as shown in equation 3). Notably if we add in interim data, then from the perspective of Ville's inequality, they are indistinguishable for testing. The methodology also allows for combining data from trials without requiring a common design -- this can easily done by deciding upon a min mu parameter for each trial, using which one can still get a valid combination of different trials with valid type I guarantees. Further, method not only captures whether an effect is statistically significant or not, it also captures evidence up till now. The language of the "implied target" of Shafer is used to make this precise, which in turn can also be used to quantify how much will the evidence change if a new study with some mu and N is added. 

 The paper also reports testing the methodology during the covid19 pandemic in two meta-analysis. One involving 7 trials, and the other involving 4 trials - considering different populations (healthcare workers, and the elderly). The trials involved testing if BCG could help with COVID19 immunity. The results are discussed while discussing issues (and recommendations) for meta-analysis design, systematic search for trials, systematic reviews for trial inclusion, data upload, and disseminating results. 

 In general, I found the paper very well written. The methodology is described very clearly, along with a glimpse of the underlying statistical tools available. The advantages and recommendations that the methodology has/implies are also discussed in detail. The underlying mathematics for testing is standard, but as far as I understand this is the first application for it in the setting considered in the paper. I would recommend the paper for acceptance.

Is the rationale for developing the new method (or application) clearly explained?

Yes

Is the description of the method technically sound?

Yes

Are the conclusions about the method and its performance adequately supported by the findings presented in the article?

Yes

If any results are presented, are all the source data underlying the results available to ensure full reproducibility?

No source data required

Are sufficient details provided to allow replication of the method development and its use by others?

Yes

Reviewer Expertise:

Machine Learning, Statistics, Conformal Prediction, Sequential Testing, Drug Discovery.

I confirm that I have read this submission and believe that I have an appropriate level of expertise to confirm that it is of an acceptable scientific standard.

# Response

**Published:** 2025-03-03

**Authors:** Judith ter Schure

**Affiliations:** CWI, The Netherlands

We apologize for this very late reply. However late, we still hope to contribute to a complete and accurate scientific literature with a Version 2 of our paper that incorporates all the very useful comments made by the reviewers. Version 2 also further illustrates the unique features of the method by extending the example ALL-IN prospective meta-analysis in the paper with a reflection on the collaboration, summary of results and references to a complete replication package.

 We thank Shubhendu for his very kind and thoughtful reflection on our paper.

 We actually like ‘ *Anytime Live and Learning Interim Meta-Analysis’ *as a separate interpretation of the ALL-IN acronym, even though this might have been a typo.

 In this paper we introduced the ALL-IN method by examples in which a smallest effect-size of interest could be set: the FDA COVID-19 vaccine game and the BCG collaboration. While this setting in ubiquitous in theory –with every power analysis setting a maximum sample size controlling type-II errors by pretending to be based on such an effect-size of minimal interest, in practice many clinical researchers have difficulty setting one. Moreover, in noninferiority settings it is even impossible to set one. One of us has discussed these two issues briefly in a discussion piece (Ter Schure, 2024).

 While the ALL-IN methods presented in our paper are optimal in both simplicity and efficiency in settings with a smallest effect-size of interest, these other settings need further research, as well as their own introduction paper. As these settings require to *learn* the betting strategy, or alternative hypothesis parameter, from the data, we might as well call it Anytime Live and Learning meta-analysis!

 ter Schure, J. (2024). Judith ter Schure’s contribution to the Discussion of ‘Safe testing’ by Grünwald, de Heide, and Koolen. *Journal of the Royal Statistical Society Series B: Statistical Methodology*, *86*(5), 1157-1159.

# Reviewer report: Reviewer response for version 1

**DOI:** 10.5256/f1000research.77953.r146413

**Published:** 2022-09-16

**Authors:** Ewelina Rogozinska

**Affiliations:** 1 MRC Clinical Trials Unit, Institute of Clinical Trials and Methodology, University College London, London, UK

The work by ter Schure and Grünwald outlines an alternative approach to evidence synthesis, aiming to include emerging evidence in real time without increasing type-1 error. The authors suggest that their ALL-IN method can “breathe life into living systematic reviews, through better and simpler statistics, efficiency, collaboration and communication”. As much as I agree with the three final points and fully support this notion, I have some doubts regarding the professed ‘simplicity’ of the proposed statistical approach, implementation, and generalizability of the method.

 Firstly, the approach has been developed and tested only in one very unusual setting (Covid-19 pandemic), in which the accumulation of evidence over a short space of time was extreme and sharing of data and collaboration was greater than witnessed in previous years. According to Heinze *et al.* (manuscript in submission) and their proposed framework of four phases of methodological research in biostatistics, the ALL-IN method would be classified as a method in a second phase of its development. Consequently, it requires further evaluation in a range of settings and refinement before it could be considered as a viable alternative to other available methods. This should be discussed in their paper, with declarations more balanced to reflect the single setting in which their method was applied.

 Secondly, contrary to the authors' claim that the introduction of terminology from game theory makes it easier to communicate the uncertainties, I am finding the sections using betting language difficult to follow. The evidence synthesis community still to some extent grapples with more standard methods of advanced evidence synthesis (Wang *et al.* BMJ 2021; 373: n736). ^1^ Thus, the introduction of new concepts (or their reintroduction) should be carefully thought through. Overall, I feel the manuscript would benefit from limiting the use of references to betting and investments to an essential minimum.

 Finally, the authors present a real-life example of their method using BCG vaccines trials for Covid-19. The presented example resembles an approach more akin to prospective individual participant data (IPD) meta-analysis than a living systematic review or also the referenced FAME approach - both relying on aggregate rather than individual participant data. The challenges associated with accessing IPD, the non-standard approach to data analysis and lack of clear proof of its benefits push this method toward “interesting” developments rather than “a new way forward”. The authors should explain more clearly how their method could help aggregate-level evidence synthesis or refocus the scope to IPD based projects. Furthermore, it would be interesting to learn how the ALL-IN compares to commonly used methods in terms of efficiency and reliability of obtained results.

 Concluding, the presented method is an interesting approach to evidence synthesis; however, at the current stage of its development, it requires further evaluation of its utility for the evidence synthesis to be able to bet on it.

Is the rationale for developing the new method (or application) clearly explained?

Yes

Is the description of the method technically sound?

Partly

Are the conclusions about the method and its performance adequately supported by the findings presented in the article?

Partly

If any results are presented, are all the source data underlying the results available to ensure full reproducibility?

No source data required

Are sufficient details provided to allow replication of the method development and its use by others?

Yes

Reviewer Expertise:

Conduct of systematic reviews and methodological reviews, IPD meta-analysis, bias in evidence synthesis, use of evidence synthesis in clinical practice guidelines,

I confirm that I have read this submission and believe that I have an appropriate level of expertise to confirm that it is of an acceptable scientific standard, however I have significant reservations, as outlined above.

## References

[1] : The methodological quality of individual participant data meta-analysis on intervention effects: systematic review. *BMJ* .2021; 10.1136/bmj.n736 10.1136/bmj.n736 PMC805422633875446

# Response

**Published:** 2025-03-03

**Authors:** Judith ter Schure

**Affiliations:** CWI, The Netherlands

We apologize for this very late reply. However late, we still hope to contribute to a complete and accurate scientific literature with a Version 2 of our paper that incorporates all the very useful comments made by the reviewers. Version 2 also further illustrates the unique features of the method by extending the example ALL-IN prospective meta-analysis in the paper with a reflection on the collaboration, summary of results and references to a complete replication package.

 We thank Ewelina Rogozinska for her review and for supporting our take on efficiency, collaboration and communication. We also thank her for expressing her concerns about our hopeful, but perhaps naïve, statements of simplicity in the setting of sequential meta-analysis. We indeed do not have any empirical evidence that what we propose is simple to understand. What we do know is that other available approaches to control type-I error rates in living systematic reviews have proven to be very difficult (Simmonds et al, 2017), hardly ever used and even, for general use, were recommended against by a scientific committee advising Cochrane on the matter in 2018 (reference below).

 Our main argument for simplicity is nicely pointed out by reviewer Shubhendu Trivedi, writing ‘ *we can simply collect the Z statistics Z_i from each trial, which can easily be combined (as shown in equation 3). Notably, if we add in interim data, then from the perspective of Ville's inequality, they are indistinguishable for testing.*’ So ALL-IN meta-analysis needs the same summary statistics as default Z-score approaches in conventional meta-analysis, and in terms of implementation that is indeed not simpler, but also not harder than conventional meta-analysis. The major point is that the procedure is the same whether the meta-analysis is the first analysis, or whether it is the 10 ^th^ update in a living systematic review, or whether it is the 100 ^th^ update in a prospective meta-analysis of interim results of ongoing trials. The latter nicely summarized by Rogozinska as ‘ *including emerging evidence in real time without increasing type-1 error*’. There is no other statistical approach in use in meta-analysis for which retaining type-1 error control is that simple*. Other methods need a maximum sample size, an alpha-spending function, a stopping rule to enforce on the trials, and we are convinced that these are considerations that are a lot less simple than what we propose.

 We have added a section to the Version 2 of the paper making explicit that this lack of sample size restrictions is a unique feature of ALL-IN meta-analysis that does not hold for any other statistical approach in use. The introduction section on Statistics now states (references in the paper):

*Bottom-up living meta-analysis*

*The lack of restrictions on sample size is a unique feature of ALL-IN meta-analysis that sets it apart from other statistical approaches to living systematic reviews , ‘adaptive’ (Tierney et al., 2021) or ‘real-time’ (Petkova et al, 2020) prospective meta-analysis and ‘metatrials’ (Van Haren et al, 2020). These other approaches require a maximum sample size or a maximum number of studies to guarantee type-I error control and interval coverage for all updates of the meta-analysis. The crucial difference with ALL-IN is that for those methods to be valid, participating in a prospective meta-analysis requires outside control over a trial’s data collection. Outside control is needed in the case of the ‘Framework for prospective adaptive meta-analysis’ (FAME) where a single sample size is set comprised of data from all ongoing trials, possibly at an interim of some of them, and single-analysis statistics is used. The consequence is that the intervals and p-values reported are only valid if never updated, so the analysis assumes that data collection stops after that point. No new analyses are valid after that maximum sample size, since all alpha is spent and possibly accumulation bias is introduced (Ter Schure & Grünwald, 2019). Similarly, outside control is needed in the operationalization of group-sequential or alpha-spending methods that need the information size relative to the final analysis (Simmonds et al, 2017), and therefore also need to enforce such maximum sample size for the reported results to be valid. Finally, even in in simulation-calibrated Bayesian approaches restrictions on interims (often) and maximum sample size (always) are imposed for the simulations to terminate. So the frequentist operating characteristics (type-I error, coverage of intervals) of the analysis are not known if trials wish to continue data collection after that maximum sample size and analyze again, or in other ways deviate from the simulated scenarios. Hence all available methods in living systematic reviews and prospective meta-analysis are by design quite top-down, “using preestablished stopping rules for safety, efficacy, futility, and harm” (Petkova et al, 2020).*

 With regard to simplicity of implementation, we have decided to extend Section 3 on Collaboration with a complete worked-out and reproducible example of an ALL-IN meta-analysis. This shows, among other things, that the simplicity of the statistics strengthens the collaborations when participating trials are faced with difficulties that would otherwise invalidate the analysis. Examples of these include, recruitment difficulties, delays in starting the trial, and meta-analysis results that might make a trial change course and directly influence the future sample size in an unforeseen way.

 We will address the first, second and final point in the order given. We hope that by proposing some adjustments to the paper in reply to each of his comments, we can convince Ewelina Rogozinska that the description of the method is technically sound (question 2, now answered by ‘Partly’) and that the conclusions about the method and its performance are adequately supported by the findings presented in the article (question 5, now answered by ‘Partly’). Hence we hope our V2 will be accepted without any further reservations.

 Firstly

 We wrote our paper from the hopeful perspective that many good things in science that came from the COVID-19 pandemic are here to stay, like an increase in prospective meta-analysis, data sharing and standardization of e.g. outcome measures and diagnostics. We also wrote our paper for an audience that agrees that they should. But there is nothing in the ALL-IN method that would prevent its use in a retrospective setting of meta-analysis based on summary statistics from published papers. The presentation based on *Z*-scores, and the worked-out example that we added in Section 3 show that, even though we favor IPD meta-analysis or collaboratively sharing summary statistics (the exact *e*-value itself for example), an approximate analysis often is already very good and flexible. We added the following text to the abstract:

 “ *Because the intention of the analysis does not change the validity of the results, the results of the analysis can change the intentions (‘optional stopping’ and ‘optional continuation’ based on the results so far). On the one hand: any analysis can be turned into a living one, or even become ‘real-time’ by updating with new trial data or even including interim data from trials that are still ongoing – without any changes in the cut-offs for testing or the method for interval estimation. On the other hand: no stopping rule needs to be enforced for the analysis to remain valid, so participating in a prospective meta-analysis does not require outside control over data collection.”*

 This characteristic of ALL-IN meta-analysis might even help further popularize these good things in science that came from the pandemic.

 Secondly

 We wrote our paper for an audience that considers sequential statistics to be complicated. We believe that is still our main audience because most clinical trials are not analyzed with interim analyses and most meta-analyses updates don’t take into account that repeated analyses invalidate both the *p*-value and the confidence intervals reported. While spending the better part of the past ten years on sequential analysis, we ourselves consider most of sequential statistics to be complicated. This is why we think that our audience would be confused if we just provided a ‘cook-book’ of ALL-IN methods and simply state that type-I error control and coverage of intervals is guaranteed, while using the same method over and over again. We believe that a strong intuition is needed for an audience to accept that this can be done without any corrections to the method. The game theory terminology ís that strong intuition.

 While absent in most of applied statistics today, this intuition has always been around in the history of probability theory (e.g. the work of Christiaan Huygens on games in the 17 ^th^ century), and sequential statistics (e.g.  George Alfred Barnard’s discussion of Wald’s probability ratio test in 1947). In the currently thriving literature on *e*-values and anytime-valid confidence intervals in mathematical statistics, the intuition of games is present almost everywhere and serves both as intuition to present existing results and as a very fruitful analogy that drives new results, see for example the overview article by Ramdas, Grünwald, Vovk and Shafer (2023). However, we agree that a good discussion is possible on whether references to betting and investments benefit intuition about statistics in general. We think they can, but as you can see from the discussion following Glenn Shafer’s JRSSA paper about is, there is plenty of disagreement. Yet for intuition about the sequential nature of ALL-IN meta-analysis, we do feel that the references to betting are essential for intuition, and the method should not be presented to readers familiar with the difficulties of sequential analysis without providing this intuition.

 Finally

 Our extension of the BCG example now shows more clearly that the ALL-IN method can be used on IPD (based on exact *e*-values), or can be used on summary statistics (in this example: the logrank Z-score and number of events). In fact, liaising with trials to share summary statistics can also be very fruitful just like in the FAME approach (e.g. sharing the exact e-value per trial). All the analyses of the BCG example can be reproduced based on openly available summary statistics. We hope Ewelina Rogozinska agrees that this illustrates how aggregate-level evidence synthesis is just as promising with ALL-IN methods as IPD based projects.

 We agree that future work should compare ALL-IN to other methods. For this introduction paper, we now focus on the settings where such a comparison is of less concern, as we write in the abstract: if the analysis changes the intentions. Take for example the situation in which a research team decides to update a meta-analysis and commit to a living systematic review, and after a first update decides to liaise with future trials in a prospective meta-analysis for future updates on interim results. In that situation there simply is no other statistical technique that guarantees type-I error control and confidence interval coverage, because ‘optional continuation’ decisions are made that are not prespecified and that no stopping rule-based method can handle. Hence ALL-IN breathes life into living systematic reviews in a way that no other method can.

 Cochrane Scientific Committee Schmid, C., Senn, S., Sterne, J., Kulinskaya, E., Posch, M., Roes, K., and McKenzie, J. (2018). Should Cochrane apply error-adjustment methods when conducting repeated meta-analyses?

 *It can be that simple in a fully subjective Bayesian analysis, but we have not seen any of those in use. Bayesian analysis in practice uses default priors (and possibly also informative priors, but never exclusively) and checks for frequentist operating characteristics by simulations.

 Aaditya Ramdas, Peter Grünwald, Vladimir Vovk, Glenn Shafer "Game-Theoretic Statistics and Safe Anytime-Valid Inference," Statistical Science, Statist. Sci. 38(4), 576-601, (November 2023)

 Glenn Shafer, Testing by Betting: A Strategy for Statistical and Scientific Communication, *Journal of the Royal Statistical Society Series A: Statistics in Society*, Volume 184, Issue 2, April 2021, Pages 407–431, https://doi.org/10.1111/rssa.12647

# Reviewer report: Reviewer response for version 1

**DOI:** 10.5256/f1000research.77953.r145459

**Published:** 2022-08-19

**Authors:** Junfeng Wang

**Affiliations:** 1 Division of Pharmacoepidemiology and Clinical Pharmacology, Utrecht Institute for Pharmaceutical Sciences, Utrecht University, Utrecht, The Netherlands

In this manuscript, ter Schure and Grünwald proposed a new form of meta-analysis, namely ALL-IN meta-analysis, which can facilitate living systematic reviews.

 The authors provided a different understanding of evidence synthesis, and a novel way of performing meta-analyses.

 The new method is illustrated in the language of betting, which is complement with the acronym of ALL-IN.

 Major comments:

1. The calculation of probability of next event in group X (e.g. in page 3, the fraction of 0.41 of COVID-19 events to occur in  the vaccine group, 0.41=70/(100+70)), is only valid when the number of participants is infinite in each group.
  When the number of participants still at risk in one group decreases, the probability of next event occurring in this group will also decrease. So the constant probability assumption may not be valid in real practice.
2. In page 14 and 15, the authors mentioned heterogeneity several times, however, the authors avoid giving a direct answer or solution to heterogeneous results from primary studies. This should be addressed or at least extensively discussed (as a limitation).
3. The COVID-19 vaccine trials are used as an example. However, all these trials are conducted recently and in a short time period (compared to other treatment). How is the generalizability of this new methods in other treatments?
4. The method is easily extended to IPD meta-analysis. How well this method can be extended to network meta-analysis of multiple treatments?
5. How can covariates, both on study level and individual level, be adjusted in this new framework?  

Minor comments:

1. In Page 7, section 1.1, the equation “70/170*170/70 + 100/170*170/100 = 1”, seems not correct, the right side should be 2. But I assume the authors forget adding something in the left side of the equation. Please check.
2. “Betting on vaccine” in this paper actually means betting on the next event will occur in the vaccine group (which means vaccine is not effective). It can be a bit misleading, since in common sense, reader may think betting on vaccine means betting on vaccine can protect participants.

Is the rationale for developing the new method (or application) clearly explained?

Yes

Is the description of the method technically sound?

Partly

Are the conclusions about the method and its performance adequately supported by the findings presented in the article?

Partly

If any results are presented, are all the source data underlying the results available to ensure full reproducibility?

No source data required

Are sufficient details provided to allow replication of the method development and its use by others?

Yes

Reviewer Expertise:

DTA meta-analysis, Clinical prediction models, Health Technology Assessment

I confirm that I have read this submission and believe that I have an appropriate level of expertise to confirm that it is of an acceptable scientific standard, however I have significant reservations, as outlined above.

# Response

**Published:** 2025-03-03

**Authors:** Judith ter Schure

**Affiliations:** CWI, The Netherlands

We apologize for this very late reply. However late, we still hope to contribute to a complete and accurate scientific literature with a Version 2 of our paper that incorporates all the very useful comments made by the reviewers. Version 2 also further illustrates the unique features of the method by extending the example ALL-IN prospective meta-analysis in the paper with a reflection on the collaboration, summary of results and references to a complete replication package.

 We thank Junfeng Wang for his thorough reading of our paper. We are especially grateful that he spotted a mistake in Section 1.1, pointed out as Minor comment 1, and was so kind to state “ *I assume the authors forget adding something*”. We indeed forgot to add the betting strategy, which we added in our Version 2 of the paper (see below at Minor comment 1). We also found a mistake ourselves in Section 1.3: there was a square root missing below equation (6), needing a division by 2 instead of 4 (which comes from the standard deviation of a Bernoulli-0.5 likelihood).

 ALL-IN can indeed provide a “ *different understanding of evidence synthesis, and a novel way of performing meta-analyses*”, as stated by Junfeng Wang. Although ALL-IN is a novel statistical approach to meta-analysis, it does provides statistical summaries for evidence synthesis that can be reported in a familiar way – e-values take the role of p-values, and anytime-valid confidence intervals the role of conventional confidence intervals. Our worked-out example illustrates this in Version 2 of the paper. Yet due to their anytime-validity, this statistical summary can be *Live* and can be the *Leading* source of information in decisions on future research, even at *INterim* stages of clinical trials. So while the statistical approach fits within mainstream understanding of evidence synthesis as an external and retrospective summary, it can also shift that understanding towards evidence synthesis as an active/involved (advising future trials/liaising with ongoing trials) and possibly prospective tool to facilitate collaboration and increase efficiency.

 In our Version 2 of the paper we have emphasized this aspect more, and moreover, stressed how the approach to prospective meta-analysis differs from other adaptive approaches to prospective analysis like the Framework for Prospective, Adaptive Meta-analysis (FAME), and Bayesian adaptive methods. The main difference is that the validity of the ALL-IN analysis does not rely on enforcing a maximum sample size or stopping rule, such that prospective collaborations can be more bottom-up rather than outsourcing decisions to an external top-down meta-analysis team. This fact needed a better illustration in Section 3 on Collaboration, so we rewrote that section by extending the discussion of our BCG-CORONA example with the results from that ALL-IN meta-analysis, thus including a completely worked-out example to the paper.

 We will address all further comments in the order given. We hope that by proposing some adjustments to the paper in reply to each of his comments, we can convince Junfeng Wang that the description of the method is technically sound (question 2, now answered by ‘Partly’) and that the conclusions about the method and its performance are adequately supported by the findings presented in the article (question 5, now answered by ‘Partly’). Hence we hope our Version 2 will be accepted without any further reservations.

 Major comments

1. We agree with Junfeng Wang that our representation of the FDA game is approximate and not exact, simplifying the story such that we could calculate betting scores */ e*-values based on the COVID-19 event counts alone (to keep it simple as an illustrative example). Following Junfeng Wang’s concerns, we will make this more explicit in Version 2 of the paper, after the brief mention halfway the first paragraph on the right side of page 3 ( *‘Most COVID-19 vaccine trials randomized large numbers of participants 50:50 vaccine:placebo such that we can assume that also throughout the trial the participants at risk stayed approximately balanced.’*). We add the following text and footnote there:
*This allows for a back-of-the-envelope calculation to reinterpret the design for the COVID-19 vaccine trials in the language of betting\footnote{Note that in Section 1 (specifically at the end of Section 1.1)* *we discuss how to exactly analyze such time-to-event data using e-values taking into account that the risk set changes after occurrence of events and censoring.* *We expect such a reanalysis to be very well approximated by the simple calculations that are used for illustration in this introduction of the FDA game, at least for large and balanced trials like the one by Pfizer/BioNtech. Even for trials that are a lot smaller, this approximation is quite good. For example, the exact logrank e-value of 1,88 that we report for the NL trial in Table 1 can be approximated using this back-of-the-envelope calculation for the e-value as folllows:*  *. In this trial only 1496 participants were 50:50 randomized (compared to the 40 thousand in the Pfizer/BioNtech trial), and* *96 events observed in the treatment and 110 events in the control group (Ter Schure et al, 2022); testing a 20% VE against a 0% VE.}*
  We do believe that our approximation stays true to the essence of these large vaccine trials. For the Pfizer/BioNtech trial, Polack et al (2020) report a 95% vaccine efficacy based on a beta-binomial model taking into account the confirmed cases per 1000 person-years of follow-up based on 8 cases in the vaccine and 162 cases in the control group. This corresponds very well to a simple calculation (1 – 8/162)*100 = 95% assuming a perfect 50:50 ratio of follow-up time or indeed an infinite number of participants in each group.
  The footnote points to existing text at the end of Section 1.1, the second paragraph of page 8 (in version 1 of the paper), where we do make a recommendation for actual use going forward. Just like a *p*-value logrank test, the *e*-value logrank test takes into account, as well-spotted by Junfeng Wang, that ‘ *when the number of participants still at risk in one group decreases, the probability of the next event occurring in this group will also decrease’*. Moreover, the exact *e*-value logrank test can be used in sequential settings in which the *p*-value logrank test fails, such as in sequential analysis in unbalanced randomized experiments (where p-value tests cannot be used based on group-sequential or alpha spending methods that rely on Gaussian approximations). As we write in the paper: *We will use the logrank Z-statistic as a running example for meta-analysis on summary statistics. For an IPD meta-analysis (on individual patient data), however, we recommend to use the exact e-value logrank test from Ter Schure et al. (2024) that is valid regardless of the randomization (e.g. 1:1 balanced or 1:2 unbalanced), the number of participants at risk, the number of events or the size of the effect.*
2. We believe we do address heterogeneity when we state the following on page 14: *‘heterogeneity in their effect sizes (e.g. one 20%, one 50%, one 25%) does not matter for their joint ability to reject the global null hypothesis of no effect in all trials.* *So for testing the global null, trials are allowed to be heterogeneous in where they are in the space of the alternative hypothesis H* *1* *= {VE: 20% ≤ VE ≤ 100%}. For estimation, however, it is not clear what the ALL-IN confidence interval is estimating if we assume that the effects in the trials are very different. Still, as a first summary, a typical effect size (Peto, 1987) might be useful if we are unable to estimate a random effects model. The development of confidence sequences for random-effects meta-analysis is a major goal for future work. We do not, however, believe that the evidence in a line of research should be monitored based on whether this interval excludes the null hypothesis, or whether the e-value corresponding to the random-effects null model does: for testing, the global null is much more natural. Waiting for a random-effect model to reach a certain threshold is counter-intuitive, since it might require many small trials to estimate the between-trial variability instead of focusing on testing the treatment effect. Moreover, the goal of rejecting the null hypothesis corresponding to this model can be quite strange. When testing a zero-effect null hypothesis, it assumes that there are true effects of harm and true effects of* *benefit among the trials and that their mean is exactly zero.* *’*
3. Junfeng Wang is right to point out that the COVID-19 vaccine trials are a very special example showing how meta-analysis can be done on interim trial results when many studies are simultaneously ongoing. As also stated in the abstract, ALL-IN meta-analysis can be performed on interim data, but does not have to. The analysis design requires no information about the number of patients in trials or the number of trials eventually included. In Section 1.2 we first give the example where we can collect a Z-statistic for each study out of K studies completed so far, so in a setting of conventional retrospective meta-analysis. What makes ALL-IN meta-analysis special is stated right after: the analysis on interim results is exactly the same. The connection to living systematic reviews is made earlier in the paper, on page 5 where we refer to Simmonds et al (2017). Conventional meta-analysis does not control type-I error rates in living systematic reviews, while ALL-IN meta-analysis does. We do see the need to make this connection more explicit: allowing updates of systematic reviews based on interim data is what “breathes life into living systematic reviews”. Also following another reviewer’s remarks, we will use our example of ALL-IN-META-BCG-CORONA in Section 3 to make more explicit what ALL-IN methods can do that is not possible with any other approach.
4. Future research should definitely focus on whether the method can be extended to network meta-analysis, but we have not conducted such research yet. We are pleased to see that the paper convinced the reviewer that the method is easily extended to IPD meta-analysis. We have added these considerations to the discussion section of the Version 2 paper.
5. Covariate adjustment is a major field of research in the literature on *e*-values and anytime-valid statistics. We propose to add the following paragraph to the discussion section in Version 2 of the paper:
*This paper introduces ALL-IN meta-analysis based on Z-score methods of meta-analysis, as introduced in standard works like by Borenstein et al. (2009).* *We mainly focus on testing rather than estimation. For testing, extensions to IPD meta-analysis based on exact e-values (rather than Z-score approximations) follow easily since both can be combined by multiplication, as shown in our ALL-IN-META-BCG-CORONA example. Within this focus on testing with type-I error control, the heterogeneity question is less explicit, since under the null-hypothesis there is no heterogeneity, as discussed in Section 3.3. In future work we will  provide more details on estimation under heterogeneity, with anytime-valid confidence intervals for the fixed-effect (singular), fixed-effects (plural) and random-effects model. Extensions to network meta-analysis seem possible, but are not yet our main direction. With regard to meta-regression, much development can be expected from work on e-values in settings of composite null-hypotheses, for which research is ongoing that extends to linear regression* *(e.g. Pérez-Ortiz et al., (2024)).* *For IPD meta-analyses that take covariates into account, analysis of randomized controlled trials can use the so-called ‘Model-X’ e-value approach.*
Pérez-Ortiz, M. F., Lardy, T., de Heide, R., & Grünwald, P. D. (2024). E-statistics, group invariance and anytime-valid testing. The Annals of Statistics, 52(4), 1410-1432.
Grünwald, P., Henzi, A., & Lardy, T. (2024). Anytime-valid tests of conditional independence under model-X. Journal of the American Statistical Association, 119(546), 1554-1565.

Minor comments

1. Junfeng is completely right that we forgot something: the betting strategy. We propose to correct the text as follows:
*For example putting 1/3 on vaccine and 2/3 on placebo: 1/3· 70/170 · 170/70 + 2/3· 100/170 ·170/100 = 1. No matter how we invest in the two outcomes, (e.g. try putting 1/2 on vaccine and 1/2 on placebo, or something different) in expectation under the null we multiply the initial investment by 1* *.*
2. This is a great comment! Indeed the ones betting on development of an effective vaccine – e.g. the NIH in funding the trials (taking ‘multiple shots on goal’ to just ‘let the chips fall’) – hope to find very few events of COVID-19 in the vaccine group of a clinical trial. We will adjust how we phrase this to eliminate this confusion in Version 2 of the paper.
